# From network analysis to experimental validation: identification of regulators of non-muscle myosin II contractility using the folded-gastrulation signaling pathway

**DOI:** 10.1186/s12860-023-00492-3

**Published:** 2023-10-11

**Authors:** Andy Zhao, Sophia Varady, Madelyn O’Kelley-Bangsberg, Vicki Deng, Amy Platenkamp, Petra Wijngaard, Miriam Bern, Wyatt Gormley, Elaine Kushkowski, Kat Thompson, Logan Tibbetts, A. Tamar Conner, David Noeckel, Aidan Teran, Anna Ritz, Derek A. Applewhite

**Affiliations:** Reed College Department of Biology, 3203 SE Woodstock Blvd, Portland, OR 97202 USA

**Keywords:** Non-muscle myosin II, Contractility, Folded gastrulation, Apical constriction, Drosophila melanogaster

## Abstract

The morphogenetic process of apical constriction, which relies on non-muscle myosin II (NMII) generated constriction of apical domains of epithelial cells, is key to the development of complex cellular patterns. Apical constriction occurs in almost all multicellular organisms, but one of the most well-characterized systems is the Folded-gastrulation (Fog)-induced apical constriction that occurs in *Drosophila*. The binding of Fog to its cognizant receptors Mist/Smog results in a signaling cascade that leads to the activation of NMII-generated contractility. Despite our knowledge of key molecular players involved in Fog signaling, we sought to explore whether other proteins have an undiscovered role in its regulation. We developed a computational method to predict unidentified candidate NMII regulators using a network of pairwise protein–protein interactions called an interactome. We first constructed a *Drosophila* interactome of over 500,000 protein–protein interactions from several databases that curate high-throughput experiments. Next, we implemented several graph-based algorithms that predicted 14 proteins potentially involved in Fog signaling. To test these candidates, we used RNAi depletion in combination with a cellular contractility assay in *Drosophila* S2R + cells, which respond to Fog by contracting in a stereotypical manner. Of the candidates we screened using this assay, two proteins, the serine/threonine phosphatase Flapwing and the putative guanylate kinase CG11811 were demonstrated to inhibit cellular contractility when depleted, suggestive of their roles as novel regulators of the Fog pathway.

**Supplementary Information:**

The online version contains supplementary material available at 10.1186/s12860-023-00492-3.

## Background

Apical constriction, the asymmetric constriction of the apical domain of polarized epithelial cells, is critical to a number of physiological processes. During vertebrate development, the cells at the hinge of the neural groove apically constrict to form the neural tube [1] while individual neural crest cells delaminate from the neural plate via apical constriction driven ingression, allowing them to migrate and differentiate into distinct structures [2]. Tissue folding and invagination, also driven by apical constriction, facilitate the internalization of mesoderm and endoderm precursor cells during gastrulation for both vertebrates and invertebrates [3, 4]. Finally, the apical constriction of cells below the top sheet of the epidermis and around the periphery of the wound allows for wound contraction and healing [5].

Apical constriction is largely driven by non-muscle myosin II (NM II) which, when coupled with the actin cytoskeleton, forms an actomyosin network which can drive contractility. The NMII holoenzyme is a hexamer consisting of two heavy chains, each of which is made up of a globular head domain where ATP hydrolysis and actin binding occurs as well as a central rod domain which facilitates oligomerization, two essential light chains (ELCs), which are thought to play structural roles, and two regulatory light chains (RLCs), which play a role in regulating the activation-inactivation cycle of the motor protein. NMII is maintained in an autoinhibited, inactive conformation due to interactions between the head tail of the molecule [6], preventing oligomerization and actin binding. Upon phosphorylation of the RLC*,* the autoinhibition is relieved and NMII heavy chain opens into an assembly-competent form, oligomerizing and associating with actin in an antiparallel orientation. Hydrolysis of ATP leads to the sliding of actin filaments and the subsequent generation of force [7].

Studies in *Drosophila* have contributed immensely to our understanding of apical constriction. In particular, decades of research on ventral furrow formation during gastrulation has pieced together the pathway from cell fate specification via transcription factors and G-protein coupled receptor (GPCR) signaling cascades, to force generation and cell shape change. Ventral furrow formation is regulated by the Folded-gastrulation (Fog) pathway, whereby binding of the secreted ligand Fog by the heterotrimeric GPCRs Mist and Smog, triggers the exchange of GDP for GTP in the G_⍺13_ subunit, Concertina (Cta), leading to its activation and subsequent disassociation from the complex [8]. Simultaneously, T48, a transmembrane protein which localizes to the apical domain, recruits a Rho Guanine Exchange Factor, RhoGEF2. Cta binds to RhoGEF2, which promotes the recruitment and activation of Rho1 [9]. Rho1 activates Rho kinase (Rok), which phosphorylates the RLC, encoded by *spaghetti squash (sqh*), in flies [6], activating the NMII complex. Additionally, Rok phosphorylates a myosin phosphatase complex, creating a conformational change which inhibits phosphatase activity [10]. With an actomyosin meshwork situated on the apical surface, ratchet-like pulses of apical constriction drive a reduction in apical surface area [11]. This contractile network pulls on apical adherens junctions, which transmit the force across sheets of cells, coordinating morphological changes across tissues.

While the Fog pathway has been elegantly pieced together through many decades of research, we wondered if there were other molecular players in the pathway that still remained unidentified. While much of our understanding of ventral furrow formation was gleaned from experiments using *Drosophila* embryos, cultured *Drosophila melanogaster* cells also serve as a useful model. Robust whole genome-wide screens, capitalizing on the sensitivity of S2 and S2R + cells to RNAi, have furthered our understanding of many developmental processes including the Fog pathway [12–17]. In fact, Mist, the long predicted GPCR that binds Fog, was uncovered in a cell-based contractility assay in S2R + cells [18]. Despite the ease and efficacy of whole genome-wide screens using *Drosophila* tissue culture cells, they remain time consuming and expensive. Furthermore, genome-wide screens produce many false positives, the validation of which wastes valuable resources and time [19, 20]. A computer-based protein–protein interactome network analysis is an attractive alternative to genome-wide screening and has been used as a discovery tool for the identification of new molecular partners [21–23]. When coupled with a robust bench assay, network analysis has proven to be a cheaper, faster replacement to whole genome-wide screening.

*Drosophila* S2R + cells recapitulate the Fog signaling pathway despite lacking the apical-basal polarity of epithelial cells. The addition of Fog-conditioned media leads to an increase in phosphorylated Sqh and the contraction of the perinuclear actomyosin network, leading to a constriction characterized by phase-dark and phase-light ruffles when imaged through phase-contrast microscopy and linear actin structures radiating from the nucleus, or “star-bursts,” when stained with fluorescently labeled phalloidin [16, 18].

We developed a computational pipeline to identify potential new regulators of NMII within the context of Fog signaling. Using a custom-built *Drosophila* interactome, which represents 233,054 experimentally-observed protein–protein interactions among 11,473 proteins, we developed three network-based methods to identify potential candidate regulators of Fog signaling. After combining the outputs of the methods and curating this ranked list of predictions to ensure that the candidates are expressed in S2R + cells, we ended with a list of 14 predicted regulators not previously associated with the Fog pathway (our *protein candidates*). We depleted these 14 candidates using RNAi in S2R + cells and performed a cellular contractility assay to elucidate the validity of their putative regulatory function. A secondary screen helped to eliminate a false positive from the initial results, leaving us with two potential novel regulators of the Fog pathway- Flapwing (Flw), a catalytic subunit (PP1c) of the Protein Phosphatase type 1 (PP1), as well as CG11811, a putative guanylate kinase. Depletion of these targets led to a decrease in the fraction of the cells that underwent contractility in response to the application of Fog-conditioned media, suggesting their role as regulators of NMII contractility.

## Results

### A computational framework for predicting potential regulators of Fog signaling

Before we describe the computational methods we used to predict candidate Fog signaling regulators, we first built a comprehensive resource of *Drosophila* protein–protein interactions from the literature. This information can be represented as a protein–protein interactome, which describes a set of proteins and connections between those proteins. An interactome is mathematically formulated as a graph, where the nodes (proteins) are connected by edges (protein–protein interactions). We combined six existing *Drosophila* resources of protein–protein interaction data to build an interactome with 11,473 nodes and 233,054 undirected edges, ignoring self-loops and mapping all proteins to FlyBase identifiers (Table 1). Each edge in the interactome may be supported by multiple databases; while DroID contributes the largest amount of data, 10,654 edges (5%) are not supported by DroID and would be missed by a single-database analysis. Each edge in the interactome includes literature citations as well as sources of experimental or other evidence. In total, the interactions are supported by 21,065 pubmed-indexed literature citations and 58 PSI-MI evidence sources [24].

**Table 1:** Data sources used to build the fly interactome

Data Source	Data Source Type	# Interactions^a^
FlyMine [25]	Data warehouse for functional genomic & proteomic datasets	278,370
DroID [26]	Experimentally detected interactions curated from literature and external databases	262,179
Mentha [27]	Experimentally detected physical interactions	45,669
MyProteinNet [28]	Protein interaction data compiled from 11 PPI databases	41,530
SignaLink 2 [29]	Protein interactions curated from literature, integration of interactions from PPI databases, and integration of transcription factors and their regulatory interactions	5,236
FlyReactome [30]	Expert-authored knowledgebase of reactions and pathways	612

^a^Accessed fall of 2017

**Fig. 1:**
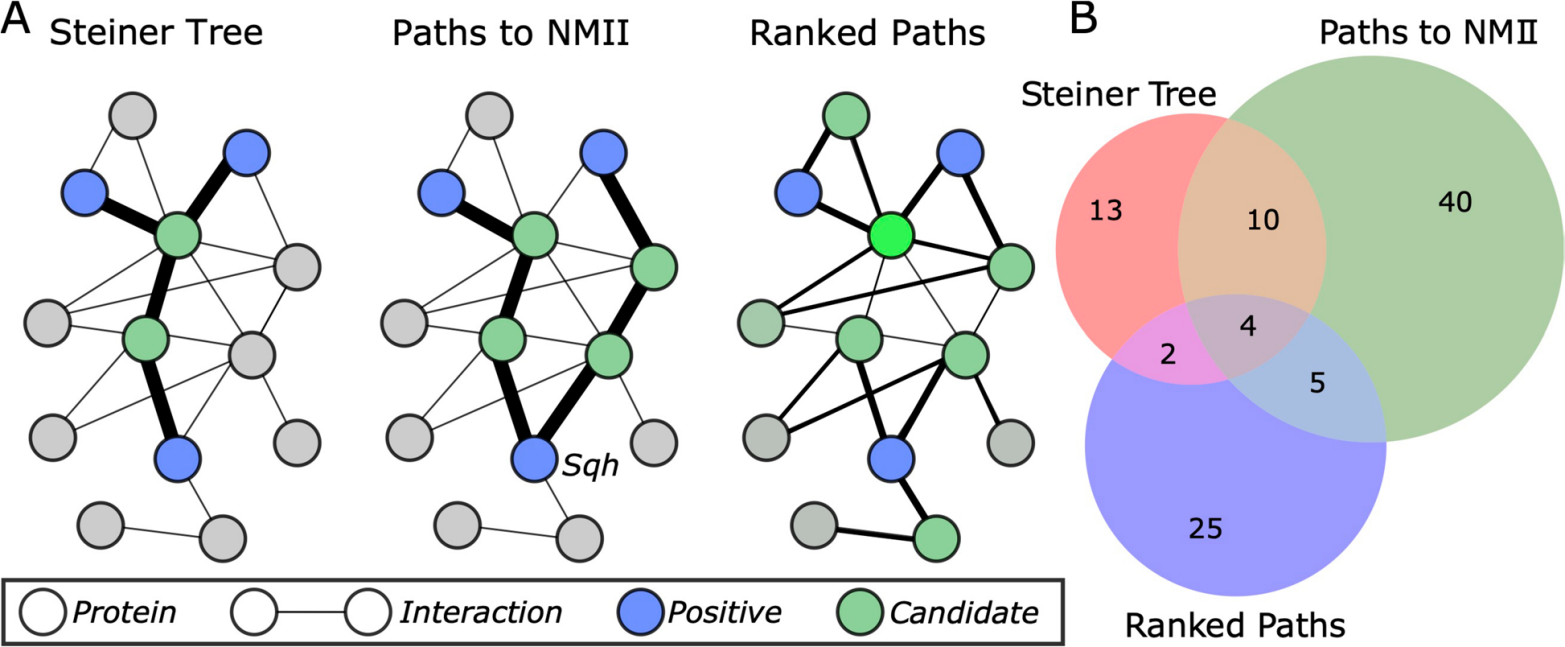
Graph algorithms used to identify protein candidates. **A** Algorithms developed to identify candidate proteins from the interactome and the positive proteins. Brighter/lighter green indicates a higher Ranked Paths score. **B** Venn diagram of protein candidates from each method; see Supplementary Table 1 for candidate list

Our computational goal was to identify candidates of NMII regulation by Fog signaling using this fly-specific interactome. We began by pooling three sets of proteins that are known to be relevant to Fog signaling [18], apical constriction (Gene Ontology 0003383), and gastrulation (Gene Ontology 0007369) to produce a list of 104 known protein regulators (called *positives;* Supplementary Table 2). Using the fly interactome, we applied three graph algorithms to identify protein candidates that are “near” the known protein regulators (Fig. 1). The Steiner Tree Approximation method aims to connect the positives with as few edges as possible; candidates are nodes that are used to connect the positives. The Paths to NMII method calculates the shortest path from each positive to Sqh; candidates are nodes that are on these paths. Finally, the Ranked Paths method calculates, for each unlabeled node, the shortest path from each positive to that node and assigns a score that is proportional to the average length of all shortest paths. All nodes in the Ranked Paths method are ranked; the nodes with a normalized score greater than 0.7 were selected as candidates (Fig. 1A). See the Methods for more details about the graph algorithms employed.

There is surprisingly little overlap among the candidates from the three methods (Fig. 1B). This is due to the differences in each method’s goal. For example, a Steiner tree will connect positives using the fewest possible non-positive nodes, so these predictions are often connected to many known positives (Supplementary Fig. 1). Calculating the paths from positives to NMII, on the other hand, may have multiple predictions on each path (though many of these paths overlap in practice, see Supplementary Fig. 2). Finally, the node score from the ranked paths approach indicates how close the node is to *all* positives, so the top predictions tend to be clustered together (Supplementary Fig. 3). A combined network of all predicted nodes, along with the adjacent positives, is shown in Supplementary Fig. 4.

Only four candidates, Cell division cycle 5 (Cdc5), Netrin-B (NetB), Spinophilin (Spn), and Ubiquitin-63E (Ubi-p63E), were identified by all three methods. NetB is not expressed in S2R + cells and thus was not pursued further [31]. Depletion of Spn is known to inhibit cellular contractility as assessed by a cellular contractility assay ([32], for details see below and Methods section) while depletion of Ubi-p63E and Cdc5 led to apoptosis preventing further assessment (unpublished data). Seventeen candidates were identified by two or more methods, and a total of 99 protein candidates were identified by any of the methods (Supplementary Table 2 and Supplementary Fig. 4). We excluded candidates that had already been shown to be a part of the Fog signaling pathway or were known Sqh interactors. As our contractility assay was performed in S2R + cells we further excluded candidates that were not expressed in these cells according to Harvard Fly RNAi database [17]. From this list of candidates 13 proteins were selected as promising for follow-up study (Table 2).

**Table 2:** Preliminary screen of computational identified candidates

*Drosophila* gene name	Symbol^a^	CG Number^b^	*Drosophila* gene name	Symbol^a^	CG Number^b^
*Ras opposite*	Rop	CG15811	*Meltrin*	Meltrin	CG7649
*14–3-3zeta*	14–3-3zeta	CG17870	*microtubule star*	mts	CG7109
*CG10347*	CG10347	CG10347	*Kinesin heavy chain*	Khc	CG7765
*Tenascin major*	Ten-m	CG5723	*corkscrew*	csw	CG3954
*flapwing*	flw	CG2096	*seven in absentia*	SinA	CG9949
*downstream of receptor kinase*	drk	CG6033	*Calcineurin B2*	CanB2	CG11217
*groucho*	gro	CG8384			

^a^Gene symbol from Flybase [33]

^B^Annotation symbol from Flybase [33]

### Preliminary screen of computational putative targets

**Fig. 2:**
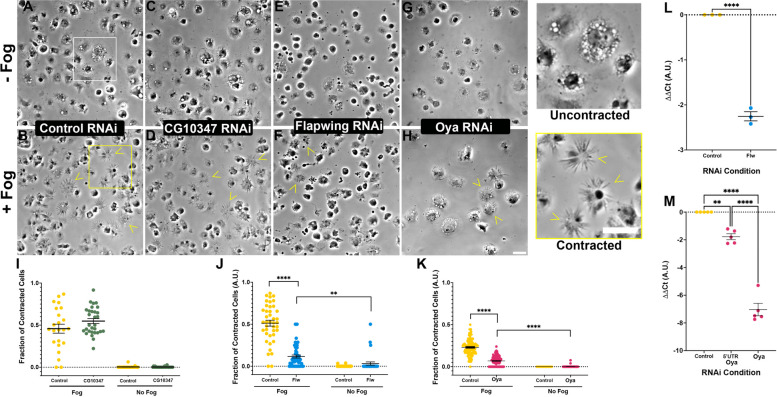
Computationally identified candidates lead to the inhibition of NMII contractility. **A**-**H** Phase-contrast images of S2R + cells treated with control (**A** and **B**), CG10374 (**C** and **D**), Flapwing (**E** and **F**), or Oya (**G** and **H**) RNAi. Cells were either perfused with control cell media (**A**, **C**, **E**, **G**) or treated with Fog-conditioned media (**B**, **D**, **F**, **H**); yellow arrows indicate cells that have contracted following Fog perfusion. The white box in (**A**) denotes uncontracted cells shown at higher magnification while the yellow box in (**B**) denotes the contracted cells shown at higher magnification (right). Scale bars 10 µm. **I**-**K** Scatter plots quantifying the fraction of contracted cells with and without Fog perfusion following RNAi treatments with CG10274 RNAi (I), Flapwing RNAi (**J**), and Oya RNAi (**K**). **I** There was no statistically significant difference between control and CG10274 RNAi cells with and without Fog treatment, however in there was an inhibition of cellular contractility following RNAi treatment with Flapwing and Oya as compared to control RNAi treated cells (****p-values < 0.0001, Student's T-test, *N* = 3). (L & M) Results from RT-qPCR indicating the efficacy of RNAi treatments for cells treated with Flapwing RNAi (**L**) and Oya (**M**). There was a statistically significant reduction in Flw mRNA as compared to control treatments (****p-value > 0.0001, Student’s t-test, *N* = 3), similarly, we observed a statistically significant reduction in Oya mRNA following RNAi treatment as compared to controls (** *p-*value = 0.0024, ****p-value > 0.0001, one-way ANOVA, *N* = 5)

We initially screened all 13 candidates (Table 2) using a cellular contractility assay [16, 34], which capitalizes on the Fog-signaling pathway [35]. Briefly, this cell-based contractility assay uses exogenously expressed Fog-myc harvested from a stable S2:Fog-myc cell line, that, when applied to S2R + cells, leads to the phosphorylation of Sqh via Rho1 signaling, leading to the contraction of cells [18, 34]. Indicative of this contractility is the observation of phase-dark and phase-light ruffling or “bonneting” when imaged by phase-contrast microscopy (Fig. 2A, B). S2R + cells were treated with RNAi for seven days and then challenged to contract. Following this initial screen we identified two candidates that failed to contract following perfusion with Fog: the PP1 catalytic subunit Flapwing (Flw) and CG10347 which, according bioinformatic queries, is putatively a member of the heat shock protein (HSP) 20 family. Both Flw and CG10347 were predicted by the Steiner and Paths to NMII methods. A second round of computational predictions that used hits from Table 2 as positive proteins revealed CG11811 (hereafter we refer to as Oya [*oya*]) which, according to bioinformatic queries and previous studies, is a guanylate kinase [36, 37]. We have subsequently named CG11811 the putative *Drosophila* guanylate kinase, Oya after the Yoruba goddess of water who is associated with fertility and acts of creation. While Oya wasn’t predicted in the first round of candidates, it has been shown to directly interact with Sqh via coimmunoprecipitation [38] (Supplementary Fig. 5).

Using an identical protocol we performed a secondary screen focused on these three candidates (Flw, CG10347, and Oya). This screen revealed that cells depleted of CG10347 still contracted, with the fraction of contracted cells following the addition of Fog not statistically different from that of control RNAi treated cells (Fig. 2A-D & I), while cells depleted of Flw and Oya failed to substantially contract following perfusion with Fog (*p* value < 0.0001, Student’s t-test, *N* = 3, *N* = 54–129 image fields per condition) (Fig. 2E-H, J & K). Given that the RNAi depletion CG10347 failed to inhibit contractility in this assay we decided to no longer pursue this candidate.

To determine the efficacy of our RNAi depletion, we performed three independent rounds of reverse transcription quantitative PCR (RT-qPCR) to determine the transcriptional abundance of either *flw* or *oya* in our RNAi treated cells. Cells treated with either *flw*, *oya,* or *oya* 5’-untranslated region (UTR) RNAi were compared against control treated cells using *elongation factor-1* (*ef1*) as an internal reference (Fig. 2L, M). The RT-qPCR data showed a statistically significant decrease in *flw* mRNA as compared to control RNAi treated samples (*p*-value < 0.001, Student’s t-test, *N* = 3) (Fig. 1L). Similarly, in cells treated with dsRNA directed against either coding region or 5’ UTR of *oya* we observed a statistically significant decrease in *oya* mRNA as compared to control RNAi treated samples albeit, the dsRNA targeting the coding region showed a far more substantial decrease (*p*-value = 0.0024, and *p*-value > 0.0001 for 5’UTR and the coding region dsRNA respectively, ANOVA, *N* = 5). Given the results of initial contractility assay and efficacy of our RNAi we decided to explore further how the depletion of Flw and Oya can lead to a decrease in NMII contractility.

#### Oya depletion affects the abundance and spatial distribution of phosphomyosin

**Fig. 3:**
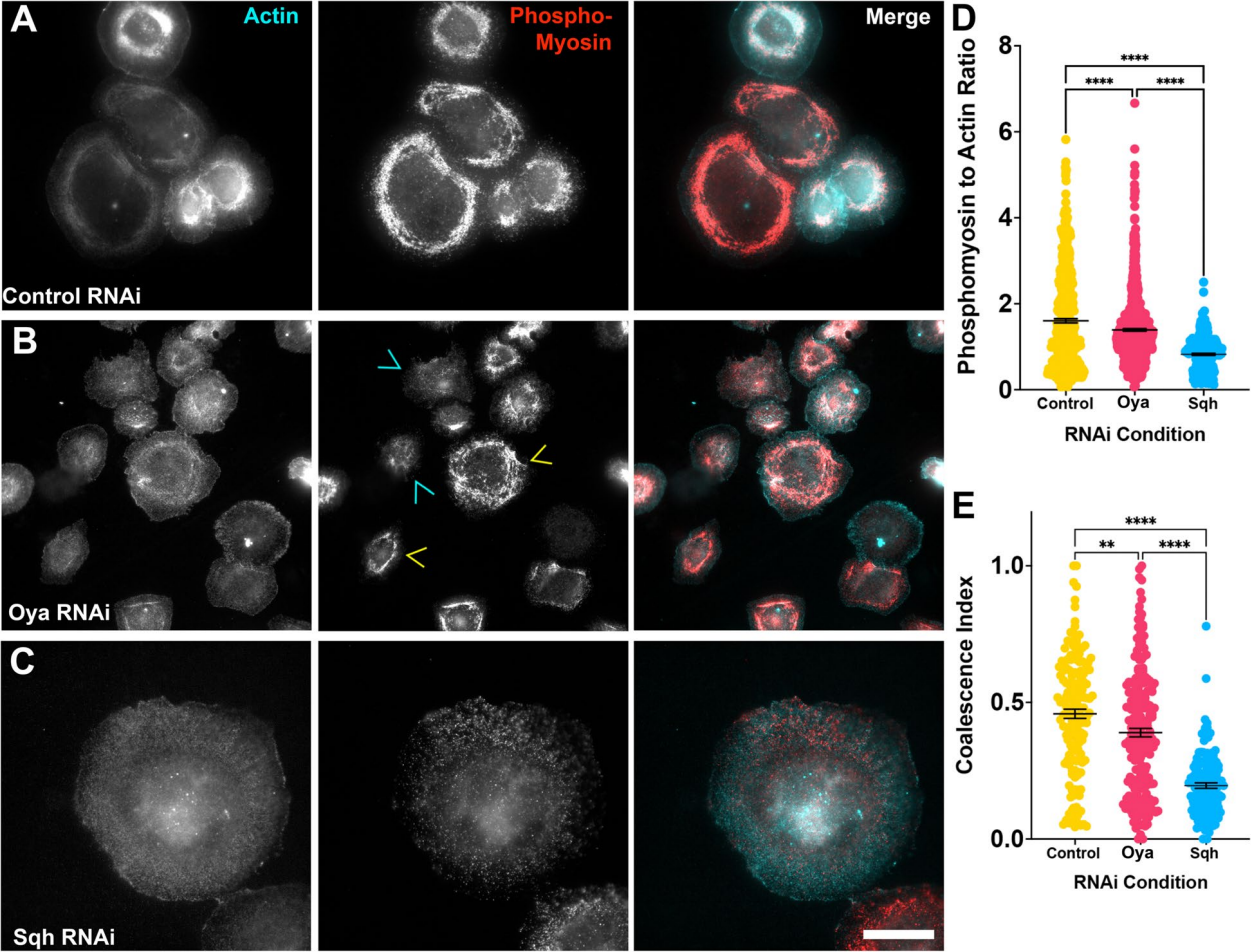
RNAi depletion of Oya disrupts the localization of phosphorylated non-muscle myosin II. **A**-**C** *Drosophila* S2R + cells stained for actin (right panel, cyan in merged image) and phosphomyosin (middle panel, red in merged image) treated with (**A**) control, (**B**) Oya, or (**C**) Sqh RNAi. Yellow arrowheads denote coalesced phosphomyosin contractile network while cyan arrowheads denote a dispersed network. Scale bar is 10 µm. **D** The quantification of the ratio of the fluorescent intensities of phoshomyosin to actin following RNAi treatments. The mean (± SEM) phosphomyosin:actin ratio in Oya depleted cells (magenta circles) was statistically significantly lower than that of control treated cells (yellow circles) but greater than that of Sqh depleted cells (blue circles) (**** *p* < 0.0001, one-way ANOVA with Tukey’s post-hoc analysis). (E) Quantification of the coalescence index measuring the degree of phosphomyosin coalescence in cells treated with control (yellow circles), Oya (magenta circles), and Sqh RNAi (blue circles). The mean (± SEM) coalescence in Oya depleted cells is less than control treated cells yet higher than Sqh depleted cells (** *p-*value = 0.00295, **** *p-*value < 0.0001, one-way ANOVA with Tukey’s post-hoc analysis *N* = 3)

In order to further probe the hypocontractility observed upon depletion of CG1181, we investigated both the abundance and spatial distribution of phosphomyosin in CG1181 depleted cells. To this end, cells were treated with either control, Oya, or Sqh RNAi for seven days and then challenged to contract by perfusion of Fog-enriched media. We then immunostained the cells with an antibody raised against a synthetic phosphopeptide mimicking phosphorylated NMII [39] as well as fluorescently-labeled phalloidin to visualize actin filaments (Fig. 3A-C). Imaging the cells by epifluorescence microscopy, we quantified the phosphomyosin staining following the Fog and RNAi treatments (Fig. 3D, E). This analysis revealed that Oya depleted cells have levels of phosphomyosin that are significantly lower than those of control treated cells but greater than that of Sqh RNAi treated cells (*p*-value < 0.00062, ordinary one-way ANOVA, *N* = 3, *N* = 289–510 cells) (Fig. 3D). Taking the fluorescence as an assay of phosphomyosin abundance, such a result suggests that, upon Fog induction, the amount of active, phosphorylated myosin is reduced in Oya depleted cells, likely contributing to cellular hypocontractility. However, Oya depletion does not completely inhibit the phosphorylation of the regulatory light chain, as evidenced by the significantly higher fluorescence values compared to Sqh depleted cells.

We then turned to probing the effects of Oya knockdown on the spatial distribution of phosphomyosin. Following Fog treatment in S2R + cells the NMII network reorganizes assembling into ordered peri-nuclear structures such as rings which are highly reminiscent of medioapical polarization observed in epithelial cells in vivo [40]. In order to quantify NMII distribution we used a previously established coalescence index [39, 41]. The higher coalescence index value the more organized (or less diffuse) the actomyosin network. This analysis reveals that Oya depletion results in a phosphomyosin distribution pattern significantly more diffuse than control RNAi treated cells yet more ordered than Sqh RNAi treated cells, mirroring the same pattern we observed in quantifying the amount of phosphomyosin (*p*-value = 0.002947, Ordinary one-way ANOVA, *N* = 3, *N* = 135–161 cells) (Fig. 3E). Thus, phosphomyosin distribution of Oya treated cells presented an “intermediate” phenotype where certain cells (Fig. 3B, yellow arrows) managed to recruit the ring of phosphomyosin seen in control treated cells while others displayed diffuse patterns of phosphomyosin (Fig. 3B, red arrows) similar to Sqh dsRNA treated cells. It also should be noted that depletion of Sqh leads to failed cytokinesis and subsequently larger cells. In addition we also imaged the distribution of EGFP-tagged Sqh in live cells following control, Oya, or Sqh dsRNA treatment (Supplemental Fig. 6A-D). Similar to our phosphmyosin staining, we observed an intermediate, diffuse pattern of Sqh-EGFP following Oya depletion whereas a clear perinuclear organization of Sqh can be observed in control depleted cells (Supplementary Fig. 6A, C and D). Depletion of Sqh led to a highly diffuse NMII pattern as expected (Supplementary Fig. 6B). Together, Oya knockdown appears to have a moderate effect on the recruitment and organization of NMII filaments, reducing the abundance of phosphomyosin while inhibiting the assembly of higher-order myosin structures which is likely contributing to the hypocontractility phenotype we observed in contractility assay.

#### Depletion of Oya may alter cytoplasmic concentrations GTP

Previous studies suggested Oya is a putative guanylate kinase due to its sequence similarity with mammalian guanylate kinases as well as its ability to phosphorylate GMP and dGMP using ATP as a phosphate donor [42]. Considering that depletion of guanylate kinases has been demonstrated to decrease intracellular concentrations of both GDP and GTP [43] as well as the centrality of GTP-mediated signaling in the Fog pathway via the Rho family of GTPases, we hypothesized that Oya’s regulation of the pathway could be due its putative role in regulating intracellular GTP levels. To test this hypothesis, we treated cells with control or Oya RNAi, or treated them with Mizoribine selective inhibitor of inosine-5'-monophosphate dehydrogenase (IMPDH) and guanosine monophosphate synthetase as a control and then fixed and stained the cells with an anti-tubulin antibody (Supplemental Fig. 7). Given the importance of GTP-tubulin to the formation of microtubules, changes to microtubule fluorescence intensity would be an indicator of cytoplasmic GTP levels. We quantified anti-tubulin fluorescence intensity and found that both treatment with Oya RNAi and Mizoribine led to a statistically significant decrease in tubulin staining as compared to control treated samples (*p*-value = 0.0001 One-way ANOVA, *N* = 38–84 cells) (Supplemental Fig. 7). Further, Oya RNAi and Mizoribine treated cells were statistically indistinguishable suggesting that RNAi Oya affects the incorporation of tubulin subunits into microtubules possibly through lowering cytoplasmic GTP levels. In terms of the NMII contractility and the Fog pathway, decreased cytoplasmic levels could affect the function of Rho family GTPases. This possibility warrants further exploration but is beyond the scope of this study. For the remainder of this study we shifted our focus to the PP1 complex component Flw.

#### Depletion of other PP1 components does not phenocopy depletion of Flapwing

**Fig. 4:**
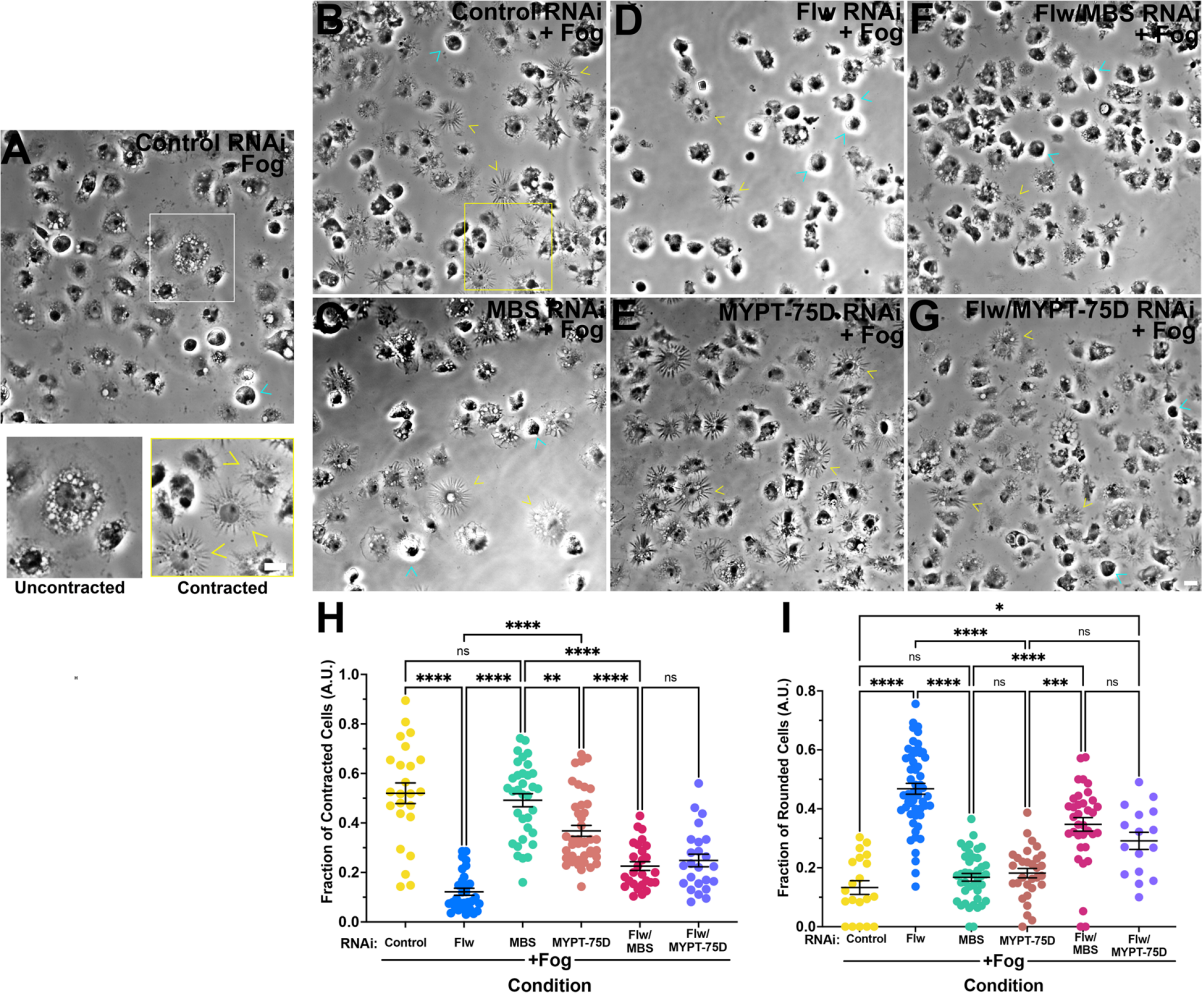
Depletion of PP1 complex has distinct effects S2R + cellular contractility. **A**-**G** Phase-contrast imaging of S2R + cells from the cellular contractility assay (**A**) in the absence of Fog or (B-G) following the perfusion of Fog. Cells were treated with (**A** and **B**) control, (**C**) MBS, (**D**) Flw, (**E**) MYPT-75D RNAi or double-depleted with (**F**) Flw and MBS, or (**G**) Flw and MYPT-75D RNAi. The white box in (**A**) denotes an uncontracted cell shown at higher magnification while the yellow box in (**B**) denotes a contracted cell shown at higher magnification.Yellow arrowheads indicate contracted cells, cyan arrowheads indicate rounded cells. Scale bars 10 µm. **H** and **I** Quantification of the mean (± SEM) fraction of contracted cells and fraction of rounded cells following treatment with control (yellow circles), Flw (blue circles), MBS (green circles), MYPT-75D (peach circles), Flw and MBS (magenta circles), and Flw and MYPT-75D RNAi (purple circles). The fraction of Flw depleted cells was statistically significantly lower than all other conditions, while depletion of MBS was no different than control RNAi treated samples. Depletion of MYPT-75D was also statistically significant from both control RNAi and Flw RNAi treated cells showing an intermediate phenotype. Double depletion of Flw and MBS and Flw and MYPT-75D also showed an intermediate phenotype, being statistically significantly different from control RNAi as well as single RNAi treatments of Flw, MBS, and MYPT-75D (**p-value = 0.0090,***p-value = 0.0004, ****p-value < 0.0001, one-way ANOVA with Kruskal–Wallis test, *N* = 3). (I) We observed a statistically significant increase in the fraction of rounded cells following Flw RNAi as compared to all other conditions. The fraction of rounded cells following MBS or MYPT-75D depletion was no different than control RNAi treated cells, while double depletion led to an intermediate cell rounding phenotype statistically significantly different from that of Flw, MBS, or MYPT-75D RNAi treated cell, as well as control cells (*p-value = 0.0417, ***p-value = 0.002, ****p-value < 0.0001, one-way ANOVA with Kruskal–Wallis test, *N* = 3)

Nurse cells mutant for *flw* exhibited hyper-contracted ring canals [44], and thus our results from the cellular contractility assay (Fig. 1E, F & J) are seemingly contradictory given that PP1 complex is commonly understood to function as phosphatase for targets such as the regulatory light chain of NMII. In order to further interrogate role of the PP1 complex in cellular contractility, we turned our attention to myosin binding subunit (MBS) and myosin phosphatase targeting subunit 75D (MYPT-75D) which are both involved in the targeting of the PP1 complex [43–45]. MYPT-75D contains a prenylation motif suggesting that it may be involved in membrane targeting while MBS lacks this prenylation [44–46]. We depleted MBS, MYPT-75D, and Flw, as well as Flw and MBS, and Flw and MYPT-75D in combination, and following our cellular contractility assay, challenged the cells to contract upon the addition of Fog (Fig. 4A-G). We again observed a statistically significant inhibition of cellular contractility following the addition of Fog in cells depleted of Flw as compared to control RNAi treated cells (*p*-value < 0.0001, ANOVA, *N* = 3–4, *N* = 50–75 cells) however, depletion of MBS failed to inhibit contractility and was no different from control RNAi treated samples (Fig. 4A-C & H). Depletion of MYPT-75D yielded mixed phenotype, with the fraction of cells undergoing contractility following the addition of Fog being statistically different from both control RNAi treated cells and that of Flw RNAi treated cells (*p*-values = 0.0389 and < 0.0001 respectively, ANOVA *N* = 3, *N* = 25–40 image fields) (Fig. 4A-E & H). Double depletion of Flw and MBS and Flw and MYPT-75D did lead to a slight rescue, with the fraction of contracted cells following Fog perfusion being statistically significant from both Flw RNAi (*p*-value = 0.004 and 0.0017, respectively ANOVA, *N* = 3, *N* = 25–40 image fields), but this rescue was incomplete as these RNAi conditions were as also statistically different from control RNAi treated cells (*p*-value < 0.0001, ANOVA, *N* = 3, *N* = 25–40 image fields) (Fig. 4A-H). These results indicate that despite potentially being a part of the same complex, the targeting subunits, MBS and MYPT-75D, and the catalytic subunit, Flw play differential roles in regulating the contractility of cells.

and I). Double depletion of Flw and MBS and Flw and MYPT-75D increased the number of rounded cells, indicative of Flw’s penetrance (Fig. 4F, G and I). These results closely mirror that of our contractility assay and again point to differences in the regulation of cellular contractility between PP1 components.

#### Flapwing, MYPT-75D, and MBS have distinct localization patterns and differentially regulate phosphomyosin distribution

**Fig. 5:**
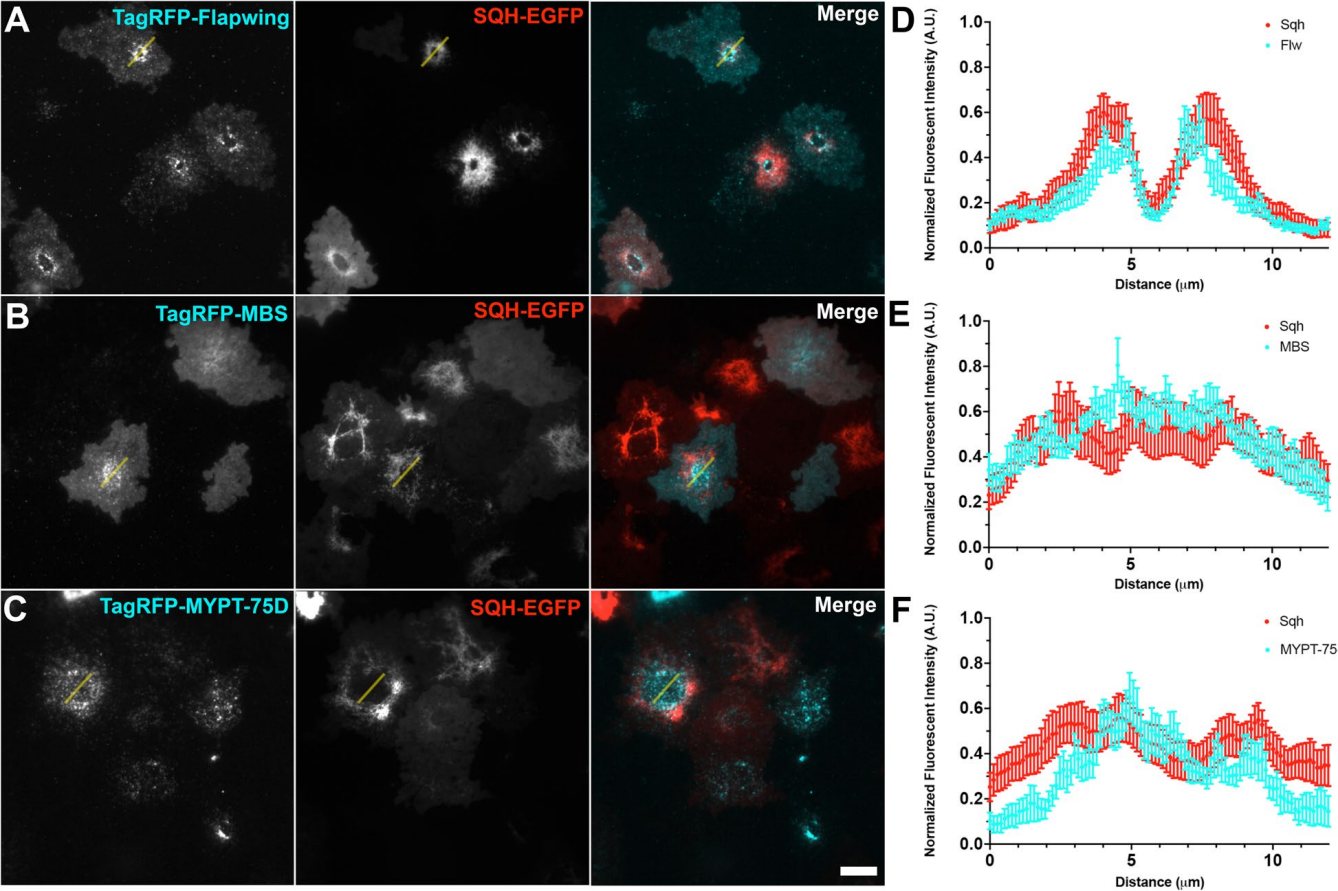
PP1 complex members have distinct localization patterns. **A**-**C** S2R + cells co-expressing TagRFP-tagged (**A**) Flapwing (left panel, cyan in merge), (B) MBS (left panel, cyan in merge), or (**C**) MYPT-75D (left panel, cyan in merge) with SQH-EGFP (middle panel, red in merge) imaged by TIRF microscopy. Yellow lines indicate a representative region of interest where line scans were taken and graphically represented in D-F. Scale bar 10 µm. **D**-**F** Line scans from cells co-expressing TagRFP-tagged (**D**) Flw, (**E**) MBS, and (**F**) MYPT-75D (all in cyan), with EGFP-Sqh (red). Graphs represent the Normalized Fluorescent Intensity of 7–10 cells per condition

NMII generated contractility relies on the concerted phosphorylation of the regulatory light chain, which results in a conformational change to the overall NMII holoenzyme allowing for oligomerization and the subsequent binding and contraction of actin filaments. Traditionally, the activity of phosphatases such as the PP1 complex, opposes this phosphorylation and maintains the NMII holoenzyme in the “closed” conformation. Previously it has been reported that MYPT-75D localizes to the cell membrane [47] while MBS can be found throughout the cytoplasm [46]. As it is likely that these spatial differences add yet another level of regulation, we sought to determine if these localization patterns are consistent in S2R + cells (Fig. 5). Consistently, we observed Flw forming a ring inside the peri-nuclear network of NMII in the center of these cells (Fig. 5A). This is in contrast to MBS, which rather than forming a distinct ring, could be found in the center of the this peri-nuclear NMII ring as an amorphous cloud (Fig. 5B), while MYPT-75D had a more global localization pattern forming a haze throughout the cytoplasm (Fig. 5C). Thus, it appears that there are differences in localization between these components of the PP1 complex which may translate to differences in the regulation of NMII contractility.

**Fig. 6:**
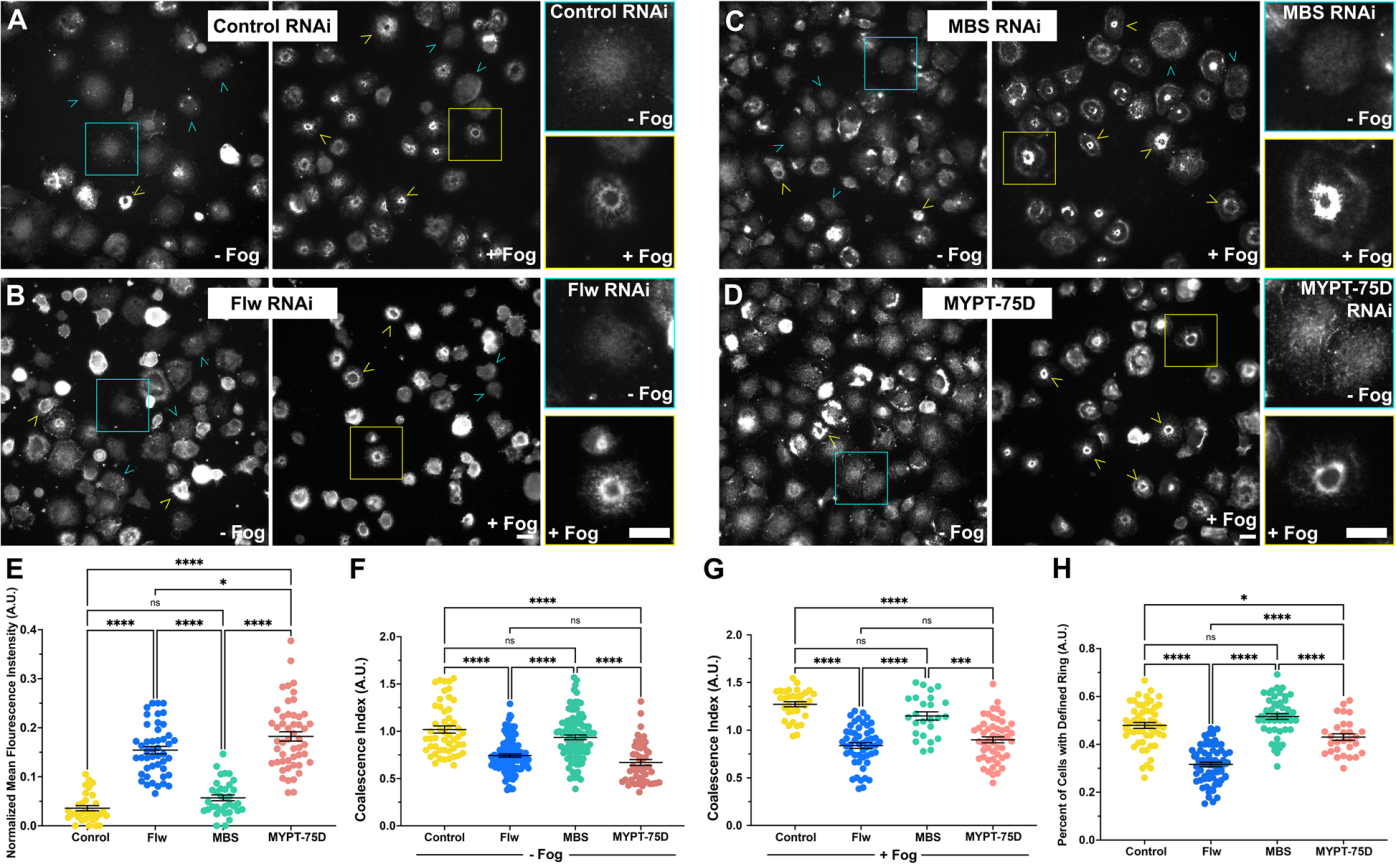
Depletion of Flw increases the amount of phosphomyosin in S2R + cells, but leads to a less organized contractile network. **A**-**D**) S2R + cells fixed and stained for phosphomyosin in the absence of Fog (left panels), or after the perfusion of Fog (right panels) following treatment with (**A**) control, (**B**) Flw, (**C**) MBS, or MYPT-75D RNAi. Yellow arrowheads denote cells with a coalesced phosphomyosin contractile network in the form of peri-nuclear rings, while cyan arrowheads indicate a more diffuse phosphomyosin network. Yellow boxes denote cells with peri-nuclear rings shown at higher magnification, while cyan boxes denote cells with a diffuse phosphomyosin network shown at higher magnification. Scale bars 10 µm. **E**–**H** Quantification of the mean (± SEM) (**E**) Normalized Fluorescence Intensity, (**F** & **G**) Coalesce Index, (**H**) Number of cells with defined rings for cells following treatment with control (yellow circles), Flw (blue circles), MBS (green circles), and MYPT-75D (peach circles). **E** Depletion of Flw and MYPT-75D led to a statistically significant increase in normalized mean phosphomyosin fluorescence intensity while the depletion of MBS was no different than that of control RNAi treated samples. **F** The coalescence index indicated that depletion of Flw and MYPT-75D were statistically indistinguishable from one another but were statistically less organized as compared to MBS and control RNAi treated samples. **G** In comparing the number of defined rings, Flw depletion led to a lower number of cells with rings as compared to all other RNAi conditions. (**p-value = ,***p-value = , ****p-value < 0.0001, one-way ANOVA with Tukey’s post-hoc analysis, *N* = 3, *N* = 29–89 image fields)

The distinct localization of PP1 complex proteins likely influences the spatiotemporal distribution of phosphorylated NMII regulatory light chain, and thus cellular contractility. To interrogate the distribution of phosphorylated NMII regulatory light chain we depleted S2R + cells of Flw, MBS, MYPT-75D or treated them with control RNAi, and then immunostained them using an anti-phosphomyosin antibody we previously used (Fig. 6A-D). Following fixation and immunostaining we imaged and quantified the mean fluorescence pixel intensity of cells depleted of Flw, MBS, MYPT-75D or treated control RNAi in the absence of Fog in order to obtain a basal phosphorylation rate (Fig. 6A-E). We found that depletion of both Flw and MYPT-75D lead to a statistically significant increase in phosphomyosin staining as compared to control or MBS RNAi conditions (*p*-value < 0.0001, ANOVA, *N* = 30–57 cells) (Fig. 6E). The observed hypocontractility, when viewed in conjunction with the increased abundance of phosphorylated myosin, suggests that Flw depleted cells might display defects in spatial or temporal regulation NMII. In order to explore this latter possibility we decided to quantify the spatial distribution of the phosphorylated regulatory light chain using our coalescence index [39, 41]. Again, the greater the coalescence index the more punctate the distribution which in turn indicates a more organized phosphomyosin network. This analysis revealed that while being statistically indistinguishable between one another, the phosphomyosin distribution of Flw or MYPT-75D was significantly more diffuse than MBS or control RNAi treated cells (*p*-value < 0.0001, ANOVA, *N* = 25–108 cells) both with and without the perfusion of Fog (Fig. 6G and H). The Fog pathway has been previously investigated in S2R + cells, where it was observed that the addition of Fog and the subsequent contractility coincided with a "purse string" structure of NMII circling the organelle-rich center domain of the cell. The contraction of this circular structure resulted in the "bonneted" morphology of S2R + cells plated on con A (Fig. 2B) [12]. Another proxy for the coalescence index is the prevalence of this phosphomyosin ring. In control treated cells, upon addition of Fog, we observed defined rings of phosphorylated myosin surrounding the central region of the cell (Fig. 6A), consistent with the literature. However, in Flw depleted cells, we saw a significant decrease in the proportion of cells displaying defined rings of phosphomyosin following Fog addition as compared to control, MBS, and MYPT-75D RNAi treated cells (*p*-value < 0.0001, one-way ANOVA, *N* = 29–89 image fields) (Fig. 6B and F). Thus, the depletion of either Flw or MYPT-75D results in an increase in the phosphorylation state of the regulatory light chain network but a decrease in its organization. Further, given some of the overlap in phenotypes, it may suggest a larger role for MYPT-75D in targeting Flw over MBS in these cells. These results also indicate that increased phosphorylation of the regulatory light chain is not enough to induce cellular contractility; it must also be spatially organized as well.

#### Loss of contractility following flapwing depletion is partially mediated through moesin activity

**Fig. 7:**
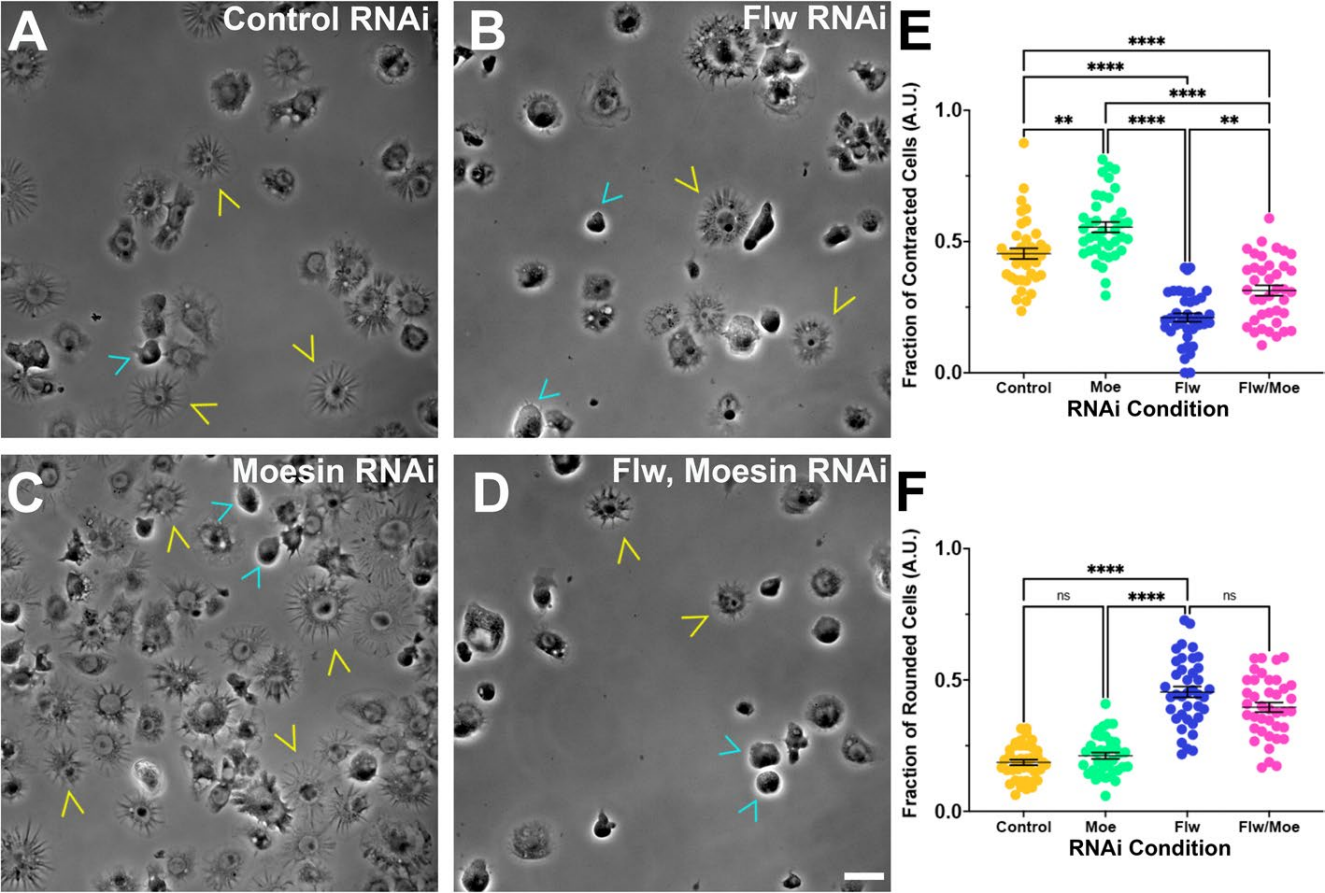
Flw regulates cellular contractility through its interaction with Moesin. **A**-**D** Phase-contrast images of S2R + cells following perfusion of Fog and treatment with (**A**) control, (**B**) Flw, (**C**) Moesin, and (**D**) Flw and Moesin RNAi. Yellow arrowheads denote contracted cells and cyan arrowheads denote rounded cells. Scale bar 10 µm. **E** and **F** Quantification of the fraction (± SEM) of (**E**) Contracted cells and (**F**) Rounded cells, treated with control (yellow circles), Moesin (mint green circles), Flw (blue circles), and Flw and Moesin (magenta circles) RNAi. **E** Depletion of Moesin led to a statistically significant increase in the fraction of contracted cells, while double depletion of Moesin and Flw only led to a partial rescue (**p-value = 0.002304,***p-value = 0.000258, ****p-value < 0.0001, one-way ANOVA with Tukey’s post-hoc analysis, *N* = 3). **F** Conversely, depletion of Moesin did not affect the fraction rounded cells we observed, while depletion of Flw led to a statistically significant increase. The double depletion of Moesin and Flw failed to rescue this cell-rounding phenotype ( ****p-value < 0.0001, one-way ANOVA with Tukey’s post-hoc analysis, *N* = 3)

Flw is known to be able to dephosphorylate and thus inactivate the Ezrin-Radixin-Moesin (ERM) protein Moesin [48]. Considering that Moesin has been implicated in driving cellular rounding [49], a phenotype observed in Flw depleted cells, we hypothesized that the phenotypes observed upon Flw knockdown could be due to an increased abundance of phosphorylated, active, Moesin. In order to test this hypothesis, S2R + cells were depleted of Moesin, Flw, and Flw and Moesin in combination or were treated with control RNAi and then were challenged to contract following perfusion of Fog (Fig. 7). Depletion of Moesin led to a hypercontractile phenotype, with a larger fraction of cells undergoing Fog-induced contractility than control RNAi treated cells (*p*-value = 0.00230, One-way ANOVA, *N* = 3). This finding may be indicative of less resistance to NMII contractility as depletion of Moesin is likely leading to inability of the cortical actin network to maintain tension. Additionally, the hypocontractile phenotype in Flw depleted cells was partially rescued by the double depletion of Flw and Moesin (*p*-value = 0.001634, One-way ANOVA *N* = 3). We also quantified the fraction of rounded cells following treatment with control, Moesin, Flw, and Moesin and Flw RNAi and observed a trend similar to our contractility assay. Moesin depleted cells were no different than control RNA depleted cells, and Flw RNAi led to stark increase in the fraction of rounded cells which was as compared to Moesin and control RNAi treated cells (*p*-value < 0.0001, One-way ANOVA, *N* = 3). However, the fraction of rounded cells failed to be rescued by depletion of Moesin and Flw in tandem. Collectively, these results indicate an antagonistic relationship between Moesin and Flw and may suggest that the hypocontractility we observed following Flw may be the result of increased cortical tension, a consequence of hyperphosphorylated Moesin. This increased tension and the loss of spatial resolution of the phosphorylated regulatory light chain of NMII is enough to inhibit contractility in our assay following Flw RNAi.

**Fig. 8:**
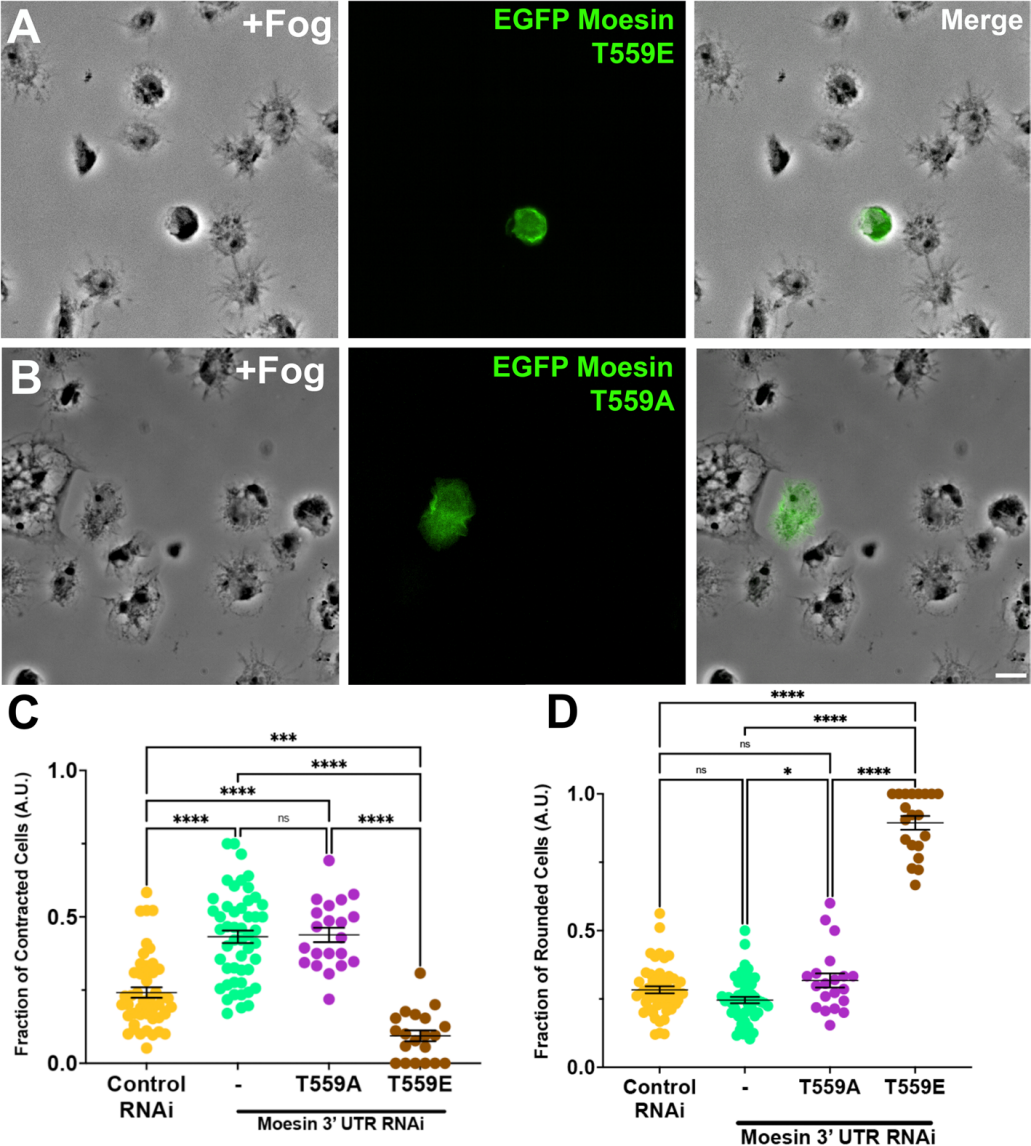
Moesin’s phosphorylation state affects cellular contractility. **A** and **B** Phase-contrast (left) and fluorescent imaging (right, green in merge) of S2R + cells following Fog perfusion treated with Moesin 3’UTR RNAi and expressing EGFP tagged (**A**) Moesin T559E or (**B**) Moesin T559A. Scale bar 10 µm. **C** and **D** Quantification of the fraction (± SEM) of (**C**) Contracted and (**D**) Rounded cells following RNAi treatment with control (yellow circles) and Moesin 3’UTR (mint green circles) RNAi. We also expressed EGFP-tagged Moesin T559A (plum circles) and Moesin T559E (brown circles) following Moesin 3’UTR RNAi. **C** The hypercontractile phenotype following Moesin 3’UTR RNAi could not be rescued by the expression of EGFP-Moesin T559A, while expression of EGFP-Moesin T559E led to a statistically significant decrease in the number of contracted cells (***p-value = 0.000174, ****p-value < 0.0001, one-way ANOVA with Tukey’s post-hoc analysis, *N* = 3). **D** Conversely, the number of rounded cells following rescue with EGFP-Moesin T559A was no different than Moesin 3’UTR RNAi alone, but expression EGFP-Moesin T559E led to a significant increase in the number of rounded cells (*p-value = 0.0254,****p-value < 0.0001, one-way ANOVA with Tukey’s post-hoc analysis, *N* = 3)

To further interrogate how the phosphorylation state of Moesin affects contractility, likely through regulating cortical tension, we generated both phosphomimetic (T559E) and non-phosphorylatable Moesin point mutants (T559A) and then tested them in our contractility assay. We depleted the endogenous pool of Moesin by using dsRNA generated against the 3’-untranslated region (UTR) of Moesin and then transiently expressed EGFP-tagged Moesin-T559A and -T559E which are refractory to this dsRNA (Fig. 8). The cells were then subjected to the aforementioned cellular constriction assay. Cells expressing phosphomimetic Moesin failed to constrict in response to Fog induction and displayed increased proportions of rounded cells, recapitulating the phenotype observed upon treatment with Flw dsRNA (Fig. 8A, C and D), while cells expressing the non-phosphorylatable form of Moesin behaved in a similar manner to Moesin depleted cells in both the fraction of contracted cells and fraction of rounded cells (Fig. 8B-D). Together, these results suggest that Flw’s regulation of cellular contraction is mediated, in part, through the increased abundance of active Moesin following Flw knockdown.

#### Phosphomyosin abundance and distribution is perturbed by phospho-mimetic moesin mutants

**Fig. 9:**
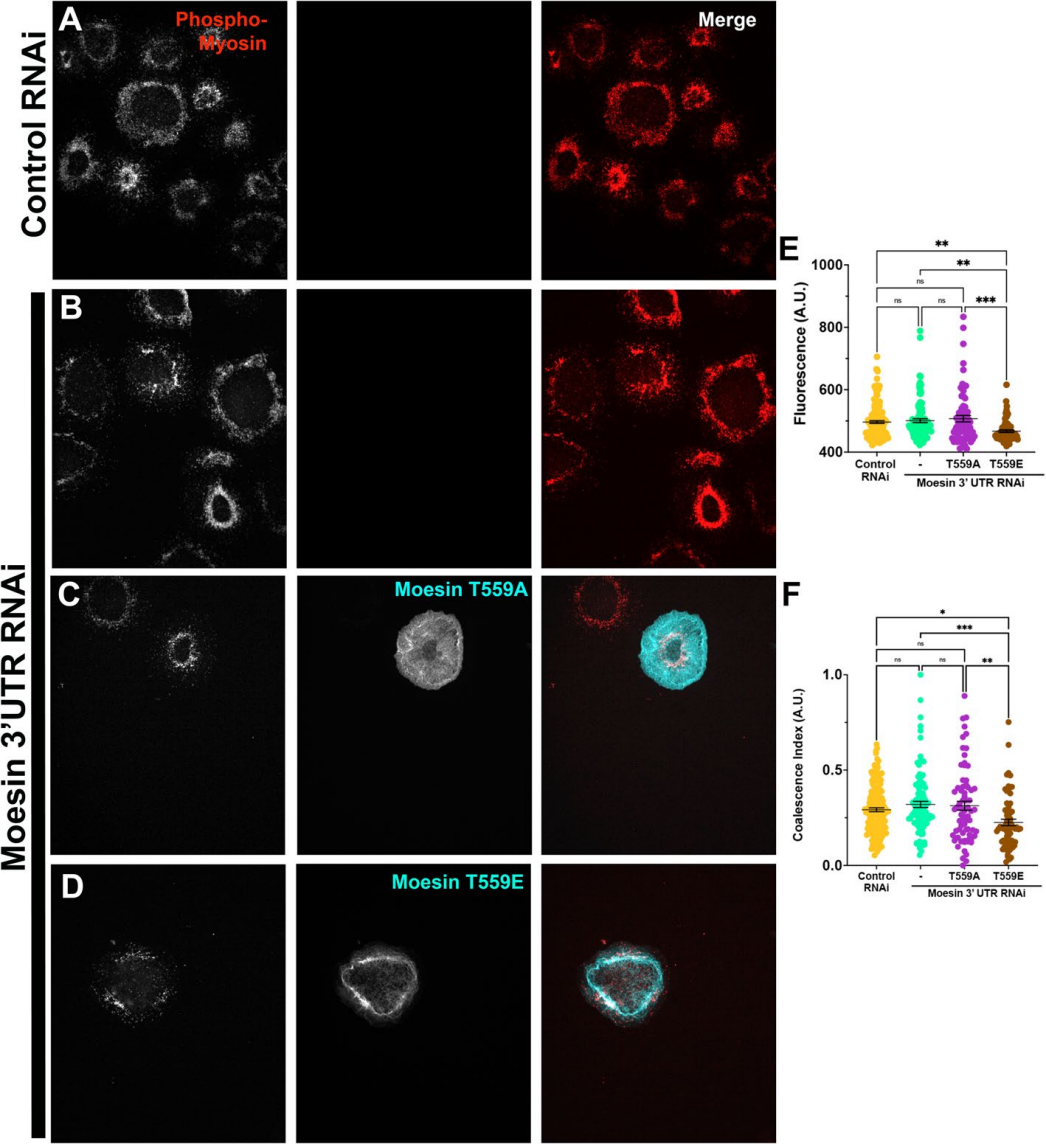
Moesin’s phosphorylation state feeds back to the phosphorylation state of NMII’s regulatory light chain. **A**-**D** TIRF images S2R + treated with (**A**) control RNAi or (**B**-**D**) Moesin 3’UTR RNAi and cells fixed and stained for phosphomyosin (left panel, red in merge). **C** and **D** Cells were also expressing EGFP-tagged (**C**) Moesin T559A (middle panel, cyan in merge) or (**D**) Moesin T559E (middle panel cyan in merge). Scale bar 10 µm. **E** and **F** Quantification of the mean (± SEM) (**C**) Fluorescence and (**D**) Coalescence Index of cells following RNAi treatment with control (yellow circles) and Moesin 3’UTR (mint green circles) RNAi. We also expressed EGFP-tagged Moesin T559A (plum circles) and Moesin T559E (brown circles) following Moesin 3’UTR RNAi. **E** Expression of Moesin T559E following Moesin 3’UTR RNAi led to a statistically significant decrease in the amount of phosphorylated NMII regulatory light chain (**p-value = 0.004123,***p-value = 0.00057, one-way ANOVA with Tukey’s post-hoc analysis, *N* = 3). Similarly, expression of Moesin T559E following Moesin 3’UTR RNAi led to a decrease in Coalescence (*p-value = 0.0149, **p-value = 0.00495,***p-value = 0.00566, one-way ANOVA with Tukey’s post-hoc analysis, *N* = 3)

Having demonstrated that the phosphorylation state of Moesin impacts cellular contractility, we then aimed to investigate if this phenotype was the result of aberrations in phosphomyosin abundance and distribution. To this end, we treated cells with either control or Moesin 3’UTR dsRNA and then transiently expressed fluorescently tagged phosphomimetic and non-phosphorylatable Moesin mutants (Fig. 9). Cells were then stimulated with Fog-conditioned before immunostaining with the aforementioned phosphomyosin antibody. This immunostaining revealed that cells depleted of Moesin displayed the characteristic pattern phosphomyosin seen in control cells and referenced previously. The expression of non-phosphorylatable Moesin-T559A did not impair the formation of these myosin rings while the expression of the phosphomimetic T559E Moesin mutant resulted in a more diffuse distribution of phosphomyosin, with atypical concentrations around the cellular cortex (Fig. 9A-D). Statistical analysis revealed a significant decrease in both the abundance and coalescence index of phosphorylated NMII in cells expressing the T559E Moesin mutant, suggesting that phosphorylated Moesin can inhibit the recruitment and higher-order assembly of phosphomyosin (Fig. 9E and F). This phenotype could be the result of Moesin’s known function as an antagonist to Rho pathway [50] as the phosphorylation of NMII by Rok is triggered by signaling from Rho1.

Considering that Flw knockdown results in increased amounts of phosphorylated, active Moesin, we would expect to see a decrease in phosphomyosin abundance in Flw depleted cells, similar to the phosphomimetic mutant expressing cells, rather than the observed increase. This apparent contradiction suggests that Flw directly regulates the phosphorylation state of NMII, as the increase in phosphomyosin abundance cannot be accounted for by regulation of Moesin alone. Together, these observations suggest that Flw regulates cellular contractility through both Moesin-mediated and Moesin-independent mechanisms.

## Discussion

The Fog signaling pathway converges on NMII contractility leading to apical constriction, a morphogenetic event that is critical to the tissue folding needed for processes such as gastrulation and neural tube formation in vertebrates, to wound healing responses and the epithelial-to-mesenchymal transition (EMT) of cells delaminating from surrounding tissues [5]. Despite being an insect specific pathway many of the molecular players involved in Fog signaling have mammalian counterparts with highly conserved functions [35, 51, 52]. Thus, the implications of this research extend beyond the Fog signaling pathway and into mammalian homeostasis and development.

Despite the intense scrutiny of the Fog pathway through the years, we wondered whether any previously unidentified proteins may also be contributing to this signaling. To address this, we built a *Drosophila* specific protein–protein interactome and developed a framework that predicts Fog signaling regulators from network-based algorithms. We have coupled this computational approach to identify potential candidates with a cell-based contractility assay which provided a means for experimental validation [16]. Using this framework, we have uncovered two new proteins involved in the regulation of Fog signaling pathway and actomyosin contractility, the phosphatase Flw, and the guanylate kinase Oya. Neither Flw nor Oya, have been previously associated with Fog signaling and our results indicate that they both may play roles in its regulation, albeit indirectly, through the regulation of the phosphorylation state of Sqh and the levels of cytoplasmic GTP levels, respectively (Fig. 2).

Oya is a putative guanylate kinase [42, 53], which is a class of enzymes that phosphorylate the nucleotide GMP into GDP using ATP as a phosphate donor. Thus, Oya could play a role in maintaining cellular concentrations of GDP. As GTP synthesis is contingent on the phosphorylation of GDP by a variety of enzymes such as nucleoside-diphosphate kinases (NDPKs), reducing levels of cellular GDP through Oya depletion could also potentially decrease the amount of cellular GTP by reducing de novo GTP biosynthesis. In support of this, a previous study demonstrated that knockdown of a guanylate kinase led to an increase of intracellular GMP and a corresponding decrease of GDP and GTP [43, 54]. In support of this, we observed a decrease in tubulin fluorescence upon Oya depletion, which was on par with Mizoribine treatment, a selective inhibitor of inosine-5'-monophosphate dehydrogenase (IMPDH) and guanosine monophosphate synthetase (Supplemental Fig. 2) suggesting that Oya depletion may reduce the amount of cytoplasmic GDP and GTP in S2R + cells. This altering of the ratio of cellular GDP/GTP could disrupt several GTP-binding signaling components of the Fog pathway, such as the G-protein coupled receptor Mist/Smog and its associated G_⍺12/13_ Concertina, as well as small GTPase Rho. Indeed, while depletion of Oya inhibited NMII contractility, led to a decrease in Sqh phosphorylation, and disrupted NMII localization (Figs. 2 and 3), in many cases these phenotypes were intermediate (Supplemental Fig. 1). These more subtle phenotypes lend further support to a global decrease in cytoplasmic concentrations rather than outright inhibition of NMII contractility. It should also be noted that the cellular contractility assay captures whether a cell contracts or not while some of the other measurements we explore in this manuscript (total phosphomyosin staining and coalescence index) capture more nuanced phenotypes. It is both an increase in phosphomyosin and the reorganization of the NMII/actin filament network that is needed for cellular contractility. We previously observed similar differences in phosphomyosin staining and reorganization upon depletion of the Rab GAP, RN-tre [39]. Further experiments, outside of the scope of this study, will need to be conducted in order to more directly test this hypothesis, but this unexpected result expands the possible mechanisms of NMII regulation.

The *Drosophila* protein phosphatase-1 (PP1) complex consists of a catalytic subunit Flw, one of two myosin phosphatase targeting subunits, MYPT-75D or MBS, which are homologous to mammalian MYPT3 and MBS85 respectively, and an M20 unit of unknown function. These proteins are highly conserved across eukaryotes [46, 47, 55]. The PP1 complex catalyzes the removal of phosphate groups from a variety of substrates. *Flw* (PP1b9C) encodes for the PP1b subtype, which is homologous to mammalian PP1b, and is sometimes referred to as PP1d. Although there is nearly 90% shared amino acid identity between PP1 complex isoforms, structural differences are conserved across homologues, suggesting evolutionarily derived differences in function [56]. Flw precipitates with Sqh and MYPT-75D in vitro [47], while mammalian MYPT3 has been shown to target PP1cδ to the regulatory light chain of myosin [45]. Flw also binds to MBS, *Drosophila mbs* mutants have been shown to have higher phosphorylated Sqh relative to controls, suggesting its role in targeting the PP1 complex to myosin [57]; and complete colocalization between MBS and Flw was observed in germ cells undergoing incomplete cytokinesis [44]. However, while MYPT-75D shows binding specificity for Flw, in vitro studies have shown that MBS lacks binding specificity for Flw as it also binds to PP1-87B [47], a PP1α subtype that was previously demonstrated to account for ∼80% of myosin phosphatase activity in third instar larvae [58]. Notably, MBS contains an inhibitory Rok phosphorylation site, while MYPT-75D does not (reviewed in [46]). Therefore, Rok directly antagonizes MBS function, but could work synergistically with the MYPT-75D/Flw complex. Evidence suggests that the MYPT regulatory subunits confer many of the differences in myosin phosphatase substrate specificity; however, the exact relationship between regulatory subunits and Flw is not well understood.

We observed distinct localization patterns of Flw, MBS, and MYPT-75D in S2R + cells respectively which adds a spacial aspect to their roles in regulating NMII contractility (Fig. 5). Previously, MBS has been described as localizing apically alongside DE-cadherin in imaginal wing discs [59]. It was also found to be apically during ventral furrow formation and during germ band extension where MBS was found to be enriched in apical junctions and in the medial pulsatile contractions along with NMII colocalizing with NMII foci [60, 61]. Flapwing also colocalizes with NMII at the ventral furrow in developing *Drosophila* embryos [62], while in the follicular epithelium of *Drosophila* egg chambers, Flw was found at the tips of basal actomyosin bundles in early stages (S6) whereas in later stages (S9) it was found dotted along the length of these actomyosin bundles [63]. MYPT-75D localized to the cell periphery in imaginal wing discs, likely due to its prenylation [47]. While three PP1 complex members coincided with the peri-nuclear organization of NMII bundles, Flw appeared more ring-like in nature, while both MBS and MYPT-75D took up more diffuse patterns in the cell center. Our cell culture system, while not epithelia in nature, has allowed us to observe these proteins at higher resolutions and these distinct localization differences may ultimately dictate phosphatase function and behavior.

*Drosophila* germ cells mutant for *flw* exhibited hyper-contracted ring canals and Flw was required for the rachet-like cycle of NMII in the basal actomyosin network of the follicular epithelial again where it is thought to counter the activity of Rok [44]. Moreover, lethal, hypomorphic alleles of *flw* can be rescued by reducing the expression of the myosin heavy chain *zipper*, or by hypomorphic alleles of *rok*, *rhogef2*, or expression of non-phosphorylatable Sqh [47], suggesting that a reduction in cellular contractility can ameliorate the reduction in Flw. Despite its connection to Sqh phosphorylation, a role Flw has yet to be explored in the context of apical constriction during Fog signaling.

Flw depleted cells exhibited hypocontractility in our cellular constriction assay which is puzzling given its role as part of PP1 complex (Figs. 2 and 4). Yang and colleagues previously demonstrated a crucial role of Flw in the regulation of Moesin family proteins, Merlin and Moesin. It was reported that Flw forms a complex with both Merlin and Moesin and activity of both proteins was altered by dephosphorylation [48]. Further, reduced Flw expression caused changes in the levels of Merlin and Moesin and was associated with defective epithelial integrity. More specifically, the reduction of Flw corresponded with an increase in Armadillo, Coracle, and F-actin expression, putatively indicating a loss of apical-basal polarity [48]. We also observed a cell rounding phenotype following Flw depletion. Again, this phenotype may be the result of Flw’s association with Moesin which plays a role in regulating cell cortex rigidity. A decrease in Flw expression leads to an increase in levels of activated Moesin, increased cell cortex rigidity and a rounded cell morphology [48, 49]. Thus, the hypocontractile phenotype we observed upon Flw depletion may be the result of increased cell cortex rigidity, countering the Fog stimulated NMII activation. In further support of this hypothesis, we observed a hypercontractile phenotype upon Moesin depletion. This may suggest that a weakened cell cortex has less resistance to NMII generated contractility and thus more easily constricts in our assay.

Our results also suggest a larger role for Moesin in the regulation of NMII constractility, with this function being somewhat independent of Moesin’s own regulation by Flw. Expression of a phosphomimetic version Moesin (T559E) inhibited Fog-stimulated cellular contractility, an overall decrease in phosphorylated Sqh, and a decrease in NMII coalescence. Moesin has been documented to antagonize Rho signaling in both *Drosophila* wing discs epithelium and LLC-PK1 epithelial cells [50], and given the centrality of Rho signaling to the Fog pathway Moesin should also be added to the growing list of Fog signaling associated proteins.

## Conclusion

We have used computational approaches combined with experimental validation and have identified two proteins previously not associated with Fog signaling. We show that depletion of either Oya or Flw results in a decrease in phosphorylated Sqh, a disruption of NMII localization and hypocontractility following Fog perfusion. Despite disrupting NMII contractility, their mechanism of action is very different, with depletion of Oya likely leading to a global decrease in GTP levels, while depletion of Flw likely affects Moesin phosphorylation and the rigidity of the cell cortex.

## Methods

### Computational methods

We applied three different network-based algorithms to predict candidate NMII regulators from the *Drosophila* interactome. Mathematically, the interactome is represented as an undirected, unweighted graph *G* = *(V,E)* with a set *V* of nodes (proteins) and a set *E* of edges (physical interactions). We also have 104 proteins known to be relevant to Fog signaling, apical constriction, or gastrulation. These relevant proteins are called *positive nodes* in the graph *G*; the rest of the nodes in *G* are unlabeled. Computationally, the algorithms aim to identify unlabeled nodes in *G* that could be predicted by regulators based on their proximity to the positive nodes in *G.*

#### Steiner tree approximation

Given a graph *G* and a set of positive nodes, a Steiner tree is a minimum weight tree that is a subgraph of *G* and includes all of the positive nodes (and possibly other unlabeled nodes). Finding a minimum Steiner tree in a graph is NP-Hard, so we implemented an approximation algorithm that is guaranteed to return a subgraph that is no more than twice the cost of the optimal Steiner tree [64]. The candidate regulators are the unlabeled nodes that are used to connect the positives (Fig. 1A and Supplementary Fig. 1).

#### Paths to NMII

The Steiner tree approximation does not explicitly focus on NMII, so we developed a shortest-paths algorithm that accounts for the proximity of NMII in the network. Dijkstra’s algorithm efficiently finds the shortest path between any two nodes in a graph. Given a graph *G* and a set of positive nodes, we calculated the shortest path from each positive node to Sqh using Dijkstra’s algorithm, totalling 104 distinct paths. The candidate regulators are the unlabeled nodes that lie on these paths (Fig. 1A and Supplementary Fig. 2).

#### Ranked paths

Finally, there may be regulators that are not near NMII but interact with many positive nodes in the graph. We calculated the shortest path from each unlabeled node to all positives, which generates a list of 104 shortest-path lengths for each unlabeled node (call these distances${d}_{1},{d}_{2},\dots , {d}_{104}$). The score for a node is the sum of the multiplicative inverses of the distances: ${\sum }_{i=1}^{104}\frac{1}{{d}_{i}}$. These scores are then normalized to sum to one. The candidate regulators are the nodes with a normalized score greater than 0.7 (Fig. 1A and Supplementary Fig. 3).

### Cell culture

For detailed instructions on *Drosophila* tissue culture please see [14, 65]. S2R + cells were cultured at 25 °C in Shields and Sang media (S&S; Sigma S8398, 0.5 g/l NaHCO3, 1 g/l yeast extract, 2.5 g/l bactopeptone) with 10% fetal bovine serum (FBS) (Thermo Fisher Scientific, Waltham, MA) and 1 × Antibiotic–Antimycotic (Life Technologies; 15,240–062. Cells were grown in cell-culture flasks and passed every 4–5 days (Rogers & Rogers, 2008). Transfections were carried out using the FuGENE HD Transfection Reagent (Promega, Madison, WI) following the manufacturer’s instructions. Briefly, FuGENE Reagent was brought to room temperature. Plasmid DNA (2 g) was added to up to 100 µl of water. FuGENE Reagent (6 µl) was added to DNA and mixed by pipetting immediately. The mixture was incubated at room temperature for 10 min. The DNA–water mixture was then added dropwise to cells that were ∼80–100% confluent in 1 ml media six-well plate. CuSO4 (1 mM) was added to cells if necessary for induction of pMT constructs. For Mizoribine treatment, cells were incubated with 290 µM Mizoribine (Sigma-Aldrich, St. Luis, MO) for 15 min prior to imaging.

### RNAi

For instructions on how to obtain dsRNA suitable for insect culture, see [14]. Cells were incubated with 1 µl of dsRNA in 1 ml of S&S media in six-well plates at 25 °C for 24 h*.* Media and dsRNA were aspirated off and replenished each day of treatment, typically 5–7 d*.* See Table 3 for primers used to generate dsRNA.

**Table 3:** T7 Primers for dsRNA

dsRNA	CG#	Forward Primer (5’ to 3’)^a^	Reverse Primer 5’ to 3’)^a^	Reference
Control (gfp)		TGGCCCACCCTCGTGA	GCGGATCTTGAAGTTCACCTTG	This study
Flw	CG2096	CTGGCCAAAGTCAACGAT	AAGTCCGGATGTGAGGACAC	[31]
Oya	CG11811	CCTTGTCCTATGCGGTCC	ATTGATGATCTTGTGGAAGTTG	[31]
5’UTR Oya	CG11811	TTTGTGAACTGATCGACGAC	TCTGTATCTTCGGAAAGTTT	This study
MBS	CG32156	CTGGCCAAAGTCAAACGAT	AAGTCCGGATGTGAGGACAC	[12]
MYPT-75D	CG6896	CTGTGAAGATCCCGAAATCA	CGTAGCAGTTCATCGTTTTC	[31]
Sqh	CG3595	GCCCGGGATCAACTTCATGTTCCTC	GTCCTTGGCACCGTGCTTAAGG	[12]
Moesin	CG10701	AGACGCTTTGTCTCCTCATCC	GAAAAGAAGCAGCAGGAGTACG	[31]
3’UTR Moesin	CG10701	GTCGTTTTGTCTCGCAGCTT	AGAGTCGGTCCTTCCTAACC	This study

^a^Primers have the T7 promoter sequence (taatacgactcactatagg) at their 5′ end

### qPCR validation

*Flapwing, oya,* and *control (gfp)* RNAi-treatments were performed as outlined above Following 6 days of treatment, cells were pelleted by centrifugation at 300 × g for 3 min, the media was decanted, and the cells were immediately placed on ice for RNA extraction. RNA was extracted using a Promega Maxwell® 16 Instrument and Promega Maxwell® 16 Low Elution Volume simplyRNA Cells Kit, according to the protocol (Promega, Madison, WI), and eluted in 30 ml nuclease-free water. After extraction, the RNA samples were snap frozen on a mixture of dry ice and 70% ethanol, and stored at -80 °C for up to 24 h. The RNA concentration was determined with a NanoDrop ND-1000. RNA quality was visualized on a 1% w/v agarose gel with 1% bleach in TAE at 60 V for one hour. RNA quality was assessed by the presence and definition of a 28S/18S band which should occur prominently at 2,000 bp, as the 28S is processed in two fragments which run similarly to the 18S band Thermo Fisher Scientific). Following RNA extraction, reverse transcription was carried out by Promega GoScript Reverse Transcription System (#A5000; Promega, Madison, WI). 3.25 μg of RNA was added to 0.5 μg of Oglio(dT)_15_ primer in nuclease water for a total volume of 5 ml. The RNA primer was incubated at 70ºC for 5 min, chilled on ice for 5 min, then spun down for 5 s. Reverse transcription reactions included 7.2 μl nuclease-free water, 4 μl GoScript 5X Reaction Buffer, 1.2 μl MgCl_2_ (1.5 mM final), 1 μl PCR nucleotide mix (0.5 mM final), 20U Recombinant RNAsin Ribonuclease Inhibitor, and 1 μl GoScript Reverse transcriptase. Thermocycling parameters were programmed accordingly: 25 °C for 5 min, 42 °C for 1 h, then 70 °C for 15 min. cDNA products were aliquoted into 4 ml portions, snap frozen on a mixture of dry ice and 70% ethanol, and stored at -80 °C for up to 24 h. Samples were subject to only one freeze–thaw before qPCR. Quantitative PCR (qPCR) primers were ordered from QIAGEN; the exact sequences are proprietary. Elongation factor 1 (EF1), was used as a reference gene for qPCR analysis. An 8-point cDNA serial dilution curve was run in triplicate to validate the efficacy of each of the three targets (Flapwing, Oya, and EF1). Promega GoTaq qPCR Master Mix (#A6001) was used for qPCR reactions, which employs a SYBR Green dye for quantification (Promega, Madison, WI). cDNA was diluted ten-fold and added separately from a master mix into plates (#ML9651, BioRad, Hercules, CA), and sealed with Microseal ‘B’ film (#MSB1001, BioRad, Hercules, CA), in a BioExpress AirClean 600 PCR Workstation hood. Reactions were plated with three replicates per dilution and a no template and no reverse transcriptase control. Reactions were incubated at 95.0 °C for 2 min and then 40 rounds of 95.0 °C for 15 s, 55.0 °C for 40 s, and 72 °C for 30 s before a fluorescence measurement. To determine melting temperatures (Tm), primers were incubated for 5 s at 0.5 °C increasing increments from 65.0 °C to 97.0 °C. Melting temperature, gene quantification and primer efficiency were determined with BioRad CFX Manager 3.1 software (BioRad, Hercules, CA). Primers were considered to be efficient within 90–110%.

### Plasmid construction

The Gateway entry clone of *Drosophila* Moesin cDNA encoding the D isoform [66] was purchased from Addgene (Watertown, MA) and using standard Gateway cloning procedures (Thermo Fisher Scientific, Waltham, MA) was inserted into pMT-His A vector with an N-terminal EGFP tag (DGRC, Bloomington, IN). The Moesin point mutations (T559A and T559E) were generated using standard PCR based site-directed mutagenesis using the following primers: 5’-CTCGCGGAGCGCCTTGTACTT-3’ and 5’-AAGTACAAGGCGCTCCGCGAG-3’ for T559A and 5’-CTCGCGGAGCTCCTTGTACTT-3’ and 5’-AAGTACAAGGAGCTCCGCGAG-3’ for T559E.

### Immunofluorescence and imaging

Concanavalin A (con A) was applied to glass bottom dishes, removed, and the dishes were left to air dry. Once dry, cells in Shang’s and Shields media were allowed to attach for 45 min. Cell culture media was removed from the attached cells, and to the Fog-treated cells, 150 µl of Shang’s and Shields media and 50 µl concentrated Fog media was added. Control cells were supplied with 200 µl of control-conditioned Shang’s and Shields media as described in [16]. After 8 min, cells were fixed with a 10% paraformaldehyde solution (Electron Microscopy Sciences, Hatfield, PA) in PEM buffer, 100 mM PIPES, 1 M MgCl_2_, 1 mM EGTA, pH to 6.8) for 15 min at room temperature. The cells were rinsed 3 × with Tris-buffered Solution (TBS; 20 mM Tris, 150 mM NaCl, pH 7.4), and cells were blocked with 5% goat serum in TBS for 10 min. The block was removed and a 1:200 dilution rabbit anti-phosphomyosin (Ser21 light chain) primary antibody (Cell Signaling Technology, Danvers, MA) in 5% goat serum in TBS was added and left to incubate at room temperature for one hour, or at 4 °C overnight. The cells were rinsed 3 × with TBS and then incubated with a solution of 1:100 anti-Rabbit Alexa Fluor 598 secondary antibody (Jackson ImmunoResearch Laboratories, Inc., West Grove, PA), with 1:100 Alexa Fluor 488 Phalloidin and 1:10,000 Hoesct (Thermo Fisher Scientific, Waltham, MA), in TBS plus 0.1 Triton-X (Sigma-Aldrich, St. Louis, MO) and incubated in the dark at room temperature for one hour. The cells were rinsed 3 × with TBS, and mounted using Dako Fluorescence Mounting Medium (Agilent, Santa Clara, California). Cells were imaged using a Nikon Eclipse Ti-E inverted microscope (Nikon, Tokyo, Japan) using a 100x/1.49NA oil immersion TIRF objective or a 40x/0.75NA objective to visualize constriction. For quantification of phosphomyosin to actin ratios (see Fig. 3D), the integrated fluorescence intensity of the anti-phosphomyosin (Ser21 light chain) antibody was divided by the corresponding integrated fluorescence intensity of the phalloidin staining. The Normalized Mean Fluorescent Intensity (see Fig. 6E) was obtained from fluorescence intensity of the anti-phosphomyosin and was calculated by taking the fluorescent intensities of each cell as defined by its phalloidin staining and normalizing the intensity by dividing by the highest intensity value. The Fluorescence Intensity (as reported in Fig. 9E) is the integrated fluorescence intensity of each cell.

### Cell contractility assay

The cell contractility assay was performed as described in (Peters et al*.*, 2018; and Platenkamp et al*.*, 2020). Briefly, After cells were allowed to attach con A covered glass-bottom dishes. Cell culture was removed and then replaced with either Fog-conditioned media or control media. The cells were then fixed and immunostained (see above). The number of constricted and relaxed cells were counted per image frame, excluding ambiguous cell states. For control and Oya RNAi conditions the rate of ambiguous cells ranged between 2–8%, while depletion of Flw led to an increase in the number of ambiguous cell states (10–20%). These results were presented as a ratio of constricted cells over total cells by treatment per frame and analyzed for statistical significance using Prism 7 by ANOVA (Graphpad, LaJolla, CA). The percentage of contracted cells in control RNAi conditions treated with Fog is approximately 30%, however we do observe some variances across experiments. To mitigate these variances we prepare large batches of Fog (which has an approximate shelf life of three months at 4C and cannot be frozen for long term storage) and use low passaged cells. Despite these efforts some variance remains.

### Coalescence index

The coalescence index was used to measure the distribution of phosphorylated NMII Sqh at Ser21 in each treatment [39]. The coalescence index was adapted from a punctate/diffuse index [41] used to compare the progression of cytochrome c dispersal across a cell during apoptosis. To perform the measurement images of cells were thresholded in ImageJ and the cell was outlined. All values for the *XY* coordinates surrounded by the outline were obtained and normalized to a scale of 0–1. The mean over the SD of the normalized values was designated the “coalescence index” and compared across conditions. High values indicate more fluorescent pixel coalescence, while low values indicate dispersed fluorescent pixels. The relative coalescence index results were analyzed for statistical significance using Prism 7 by ANOVA (Graphpad, LaJolla, CA).

### Data availability

All data, data sets, and analyses are available upon reasonable request from the corresponding authors D.A.A. and A.R. The computational data (including the code for predicting candidates and generating networks) and interactive networks are available in the public GitHub repo https://github.com/Reed-CompBio/nmii-contractility-regulators.

### Supplementary Information

**Additional file 1: Supplementary Table 1.** Positive proteins used for the computational method. Below is a list of the three sets of proteins used as positives for the computational method. The three protein sets were combined into one set of positives for analysis. **Supplemental Table 2.** Predicted proteins from each computational method. Below is a list of the predicted protein candidates by computational method. This list does not include positive proteins that were captured by the methods.

**Additional file 2: Supplementary Figure 1.** Network visualization of proteins predicted by the Steiner tree algorithm. The Steiner tree method aims to connect all the positive proteins by using as few interactions (and intermediate nodes) as possible. Gray nodes are positive proteins and red nodes are predicted proteins. **Supplementary Figure 2.** Network visualization of proteins predicted by calculating paths to NMII. This method calculates the shortest path from each positive protein to NMII. Gray nodes are positive proteins and green nodes are predicted proteins. **Supplementary Figure 3.** Network visualization of proteins predicted by the ranked paths method. The ranked paths method aims to find proteins that are close to many positive proteins. The predicted proteins are shown in blue; for context, the positive Sqh is shown in gray. **Supplementary Figure 4.** Combined network of predictions from all three methods. Node size indicates the number of methods that found the predicted protein (large white nodes indicate proteins found by all three methods). Gray: positive (known) proteins; red: proteins predicted by only the Steiner method; green: proteins predicted by only paths to NMII; blue: proteins predicted by only the ranked paths approach; yellow: proteins predicted by Steiner & paths to NMII; cyan: proteins predicted by paths to NMII and ranked paths; magenta: proteins predicted by Steiner and ranked paths methods. The number of each color corresponds to the Venn diagram in Figure 2 of the main text. **Supplementary Figure 5.** Oya’s immediate neighbors in the network. Oya’s direct interacting partners are shown along with any interactions among those neighbors. The network includes the positive Sqh (gray) and two Ubiquitinases (green) that are found by at least one of the three algorithms. **Supplemental Figure 6.** RNAi Depletion of Oya leads to a disruption of NMII organization. (A-D) Live-cell TIRF imaging of *Drosophila* S2R+ cells expressing EGFP-tagged Sqh following treatment with (A) control, (B) Sqh, and (C & D) Oya RNAi. Scale bar 10 µm. **Supplemental Figure 7.** RNAi depletion of Oya leads to a decrease in tubulin fluorescence. (A-C) S2R+ cells fixed and stained with anti-alpha tubulin antibody following treatment with (A) control RNAi, (B) Oya RNAi, or (C) 290µM Mizoribine. Images are presented using the same gray scale for direct comparison. Scale bar is 10 µm. (D) Quantification of the mean (± SEM) of the integrated tubulin fluorescence intensity for control RNAi (dark blue circles), Oya RNAi (medium blue circles), and 290 µM Mizoribine (cyan blue circles). There was a statistically significant decrease in integrated tubulin fluorescence following Oya RNAi treatment or treatment with Mizoribine as compared to control RNAi treated cells (*p-value = 0.0143,****p-value <0.0001, one-way ANOVA with Tukey’s post-hoc analysis, N = 43-84 cells).

## Data Availability

All data and materials pertinent to this study will be made available upon reasonable request.
