# Supplementary material for: From network analysis to experimental validation: identification of regulators of non-muscle myosin II contractility using the folded-gastrulation signaling pathway
Source: BMC Mol Cell Biol. 2023 Oct 11;24:32. doi: 10.1186/s12860-023-00492-3 (PMC10568788; doi:10.1186/s12860-023-00492-3)
Supplement: Supplementary file 2 — Additional file 2: Supplementary Figure 1. Network visualization of proteins predicted by the Steiner tree algorithm. The Steiner tree method aims to connect all the positive proteins by using as few interactions (and intermediate nodes) as possible. Gray nodes are positive proteins and red nodes are predicted proteins. Supplementary Figure 2. Network visualization of proteins predicted by calculating paths to NMII. This method calculates the shortest path from each positive protein to NMII. Gray nodes are positive proteins and green nodes are predicted proteins. Supplementary Figure 3. Network visualization of proteins predicted by the ranked paths method. The ranked paths method aims to find proteins that are close to many positive proteins. The predicted proteins are shown in blue; for context, the positive Sqh is shown in gray. Supplementary Figure 4. Combined network of predictions from all three methods. Node size indicates the number of methods that found the predicted protein (large white nodes indicate proteins found by all three methods). Gray: positive (known) proteins; red: proteins predicted by only the Steiner method; green: proteins predicted by only paths to NMII; blue: proteins predicted by only the ranked paths approach; yellow: proteins predicted by Steiner & paths to NMII; cyan: proteins predicted by paths to NMII and ranked paths; magenta: proteins predicted by Steiner and ranked paths methods. The number of each color corresponds to the Venn diagram in Figure 2 of the main text. Supplementary Figure 5. Oya’s immediate neighbors in the network. Oya’s direct interacting partners are shown along with any interactions among those neighbors. The network includes the positive Sqh (gray) and two Ubiquitinases (green) that are found by at least one of the three algorithms. Supplemental Figure 6. RNAi Depletion of Oya leads to a disruption of NMII organization. (A-D) Live-cell TIRF imaging of Drosophila S2R+ cells expressing EGFP-tagged Sqh following tr [file 12860_2023_492_MOESM2_ESM.docx]

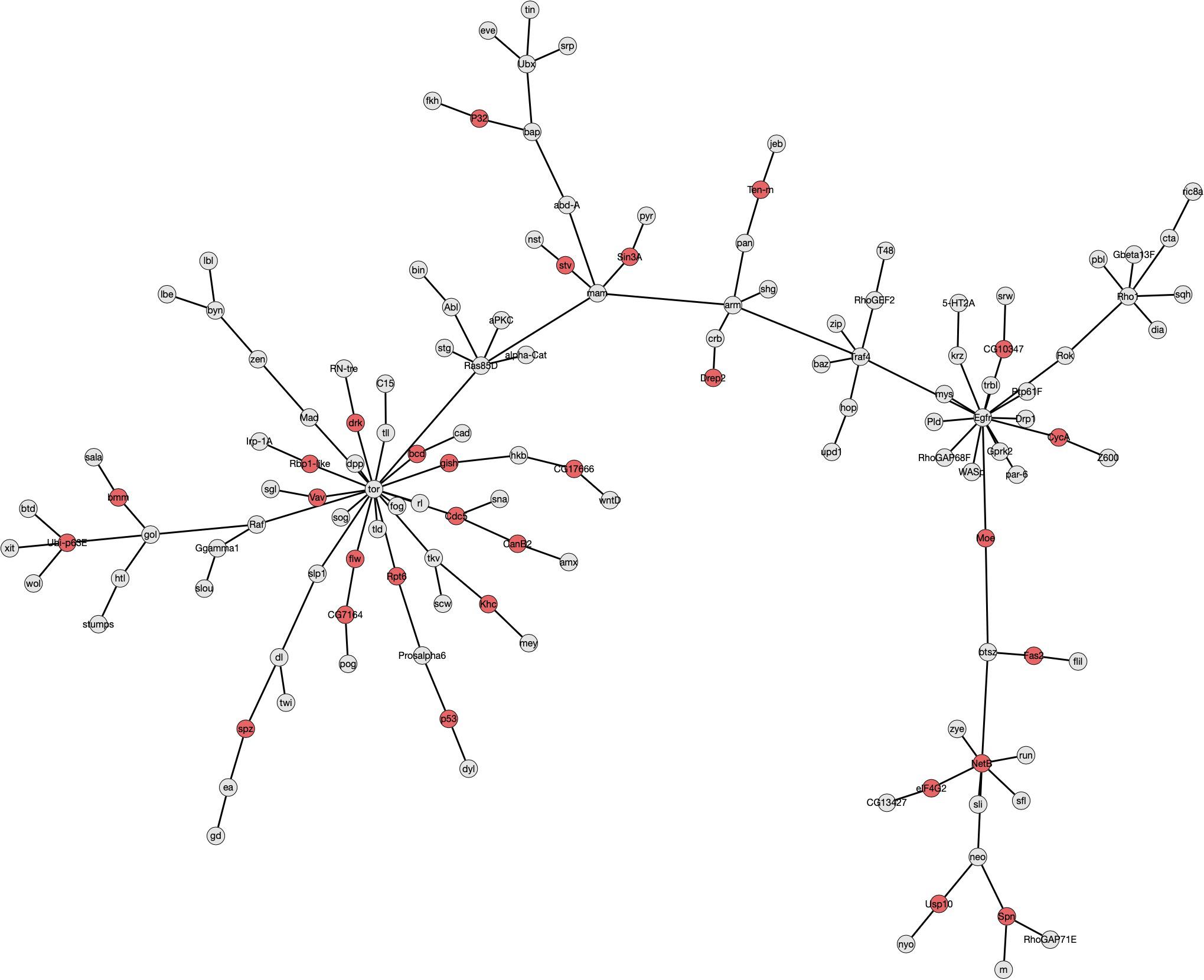


**Supplementary Figure 1. Network visualization of proteins predicted by the Steiner tree algorithm.** The Steiner tree method aims to connect all the positive proteins by using as few interactions (and intermediate nodes) as possible. Gray nodes are positive proteins and red nodes are predicted proteins.


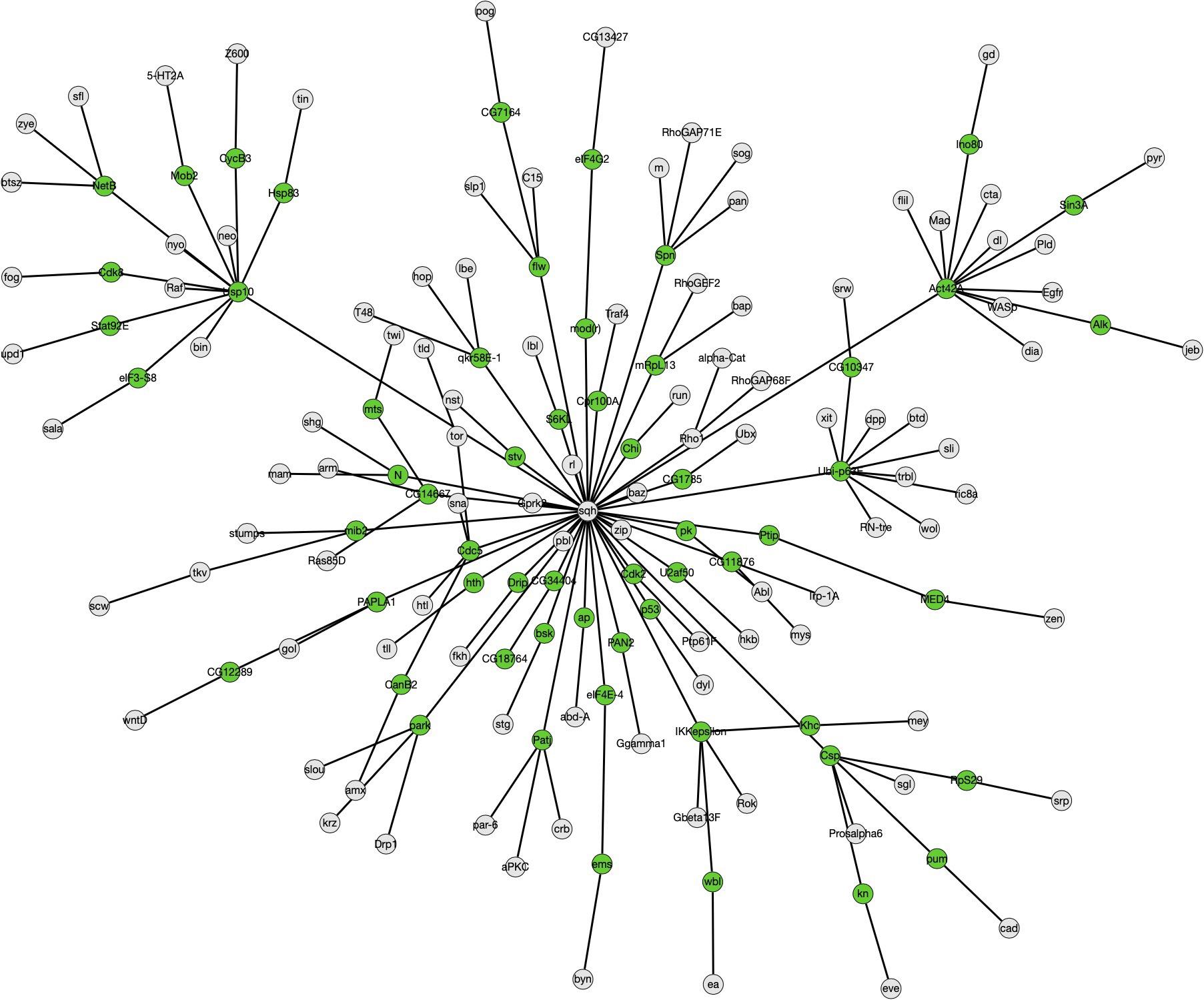


**Supplementary Figure 2. Network visualization of proteins predicted by calculating paths to NMII.** This method calculates the shortest path from each positive protein to NMII. Gray nodes are positive proteins and green nodes are predicted proteins.


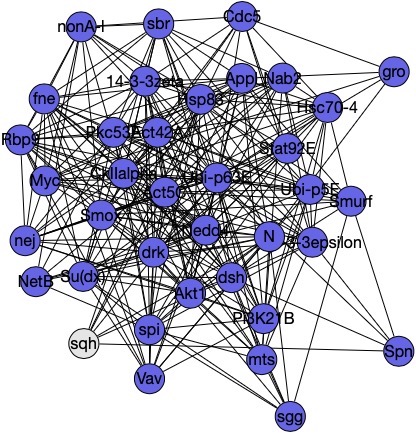


**Supplementary Figure 3. Network visualization of proteins predicted by the ranked paths method.** The ranked paths method aims to find proteins that are close to many positive proteins. The predicted proteins are shown in blue; for context, the positive Sqh is shown in gray.


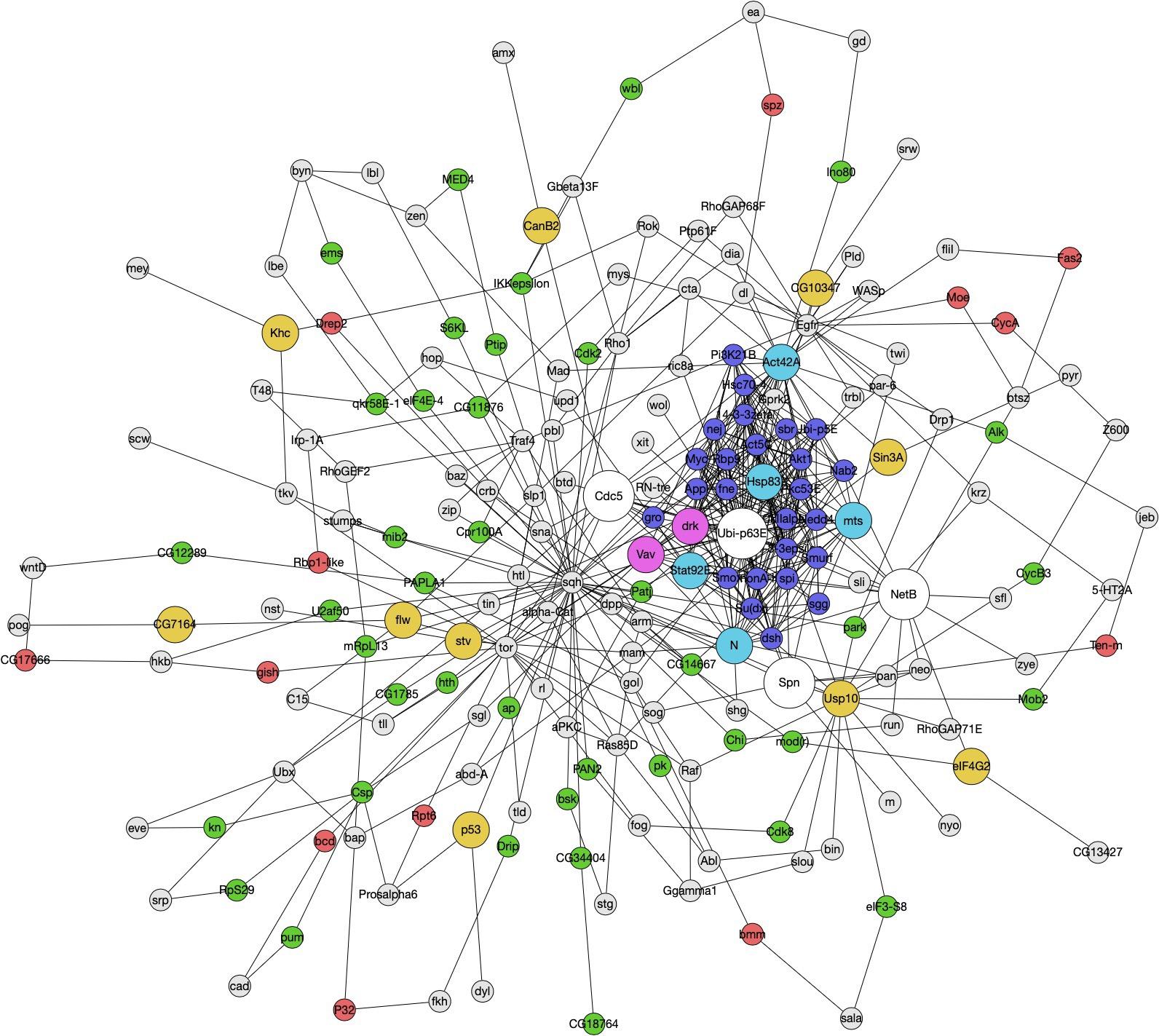


**Supplementary Figure 4. Combined network of predictions from all three methods.** Node size indicates the number of methods that found the predicted protein (large white nodes indicate proteins found by all three methods). Gray: positive (known) proteins; red: proteins predicted by only the Steiner method; green: proteins predicted by only paths to NMII; blue: proteins predicted by only the ranked paths approach; yellow: proteins predicted by Steiner & paths to NMII; cyan: proteins predicted by paths to NMII and ranked paths; magenta: proteins predicted by Steiner and ranked paths methods. The number of each color corresponds to the Venn diagram in Figure 2 of the main text.


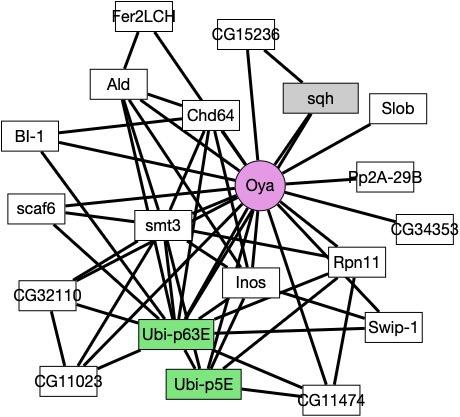


**Supplementary Figure 5. Oya’s immediate neighbors in the network.** Oya’s direct interacting partners are shown along with any interactions among those neighbors. The network includes the positive Sqh (gray) and two Ubiquitinases (green) that are found by at least one of the three algorithms.


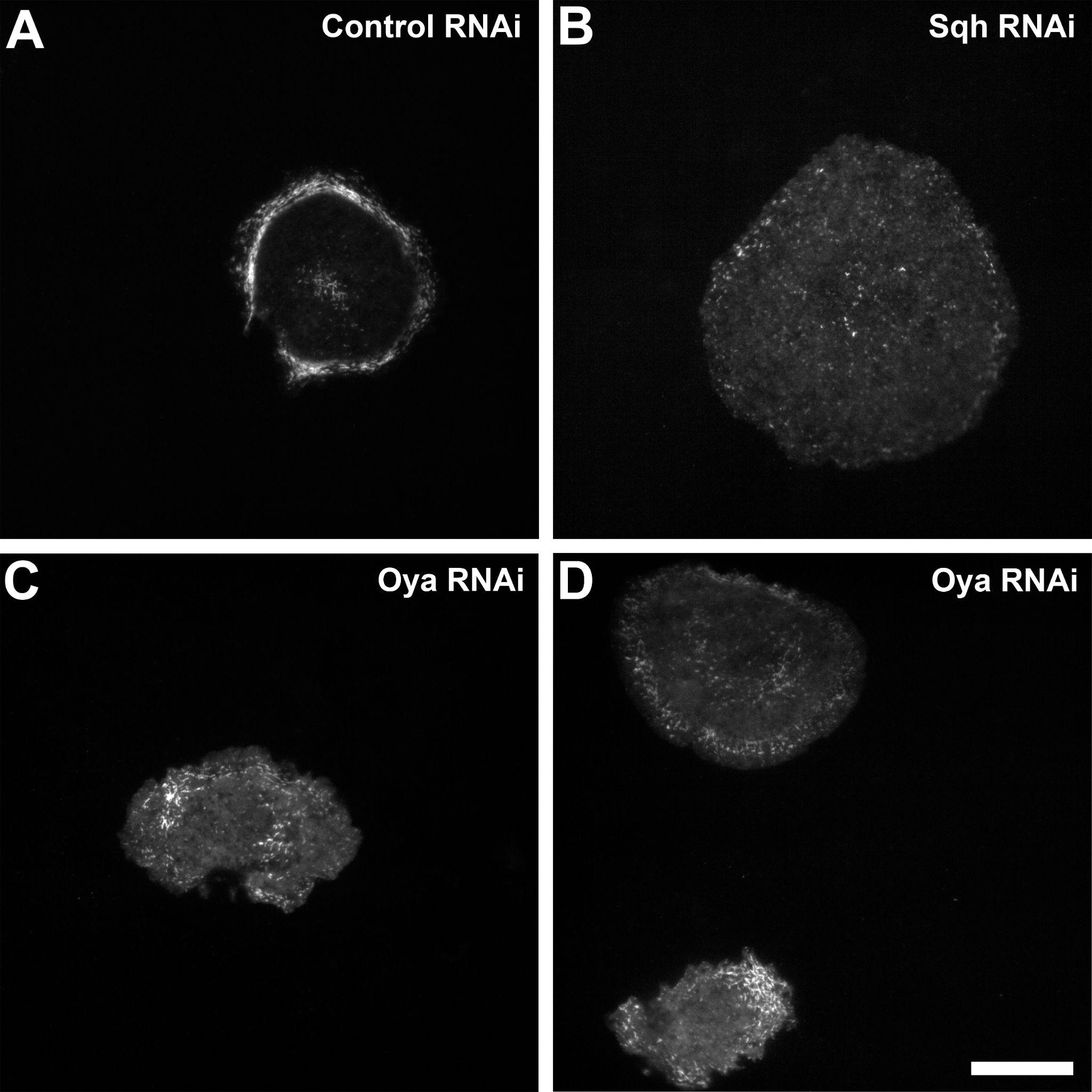


**Supplemental Figure 6. RNAi Depletion of Oya leads to a disruption of NMII organization.** (A-D) Live-cell TIRF imaging of *Drosophila* S2R+ cells expressing EGFP-tagged Sqh following treatment with (A) control, (B) Sqh, and (C & D) Oya RNAi. Scale bar 10 µm.


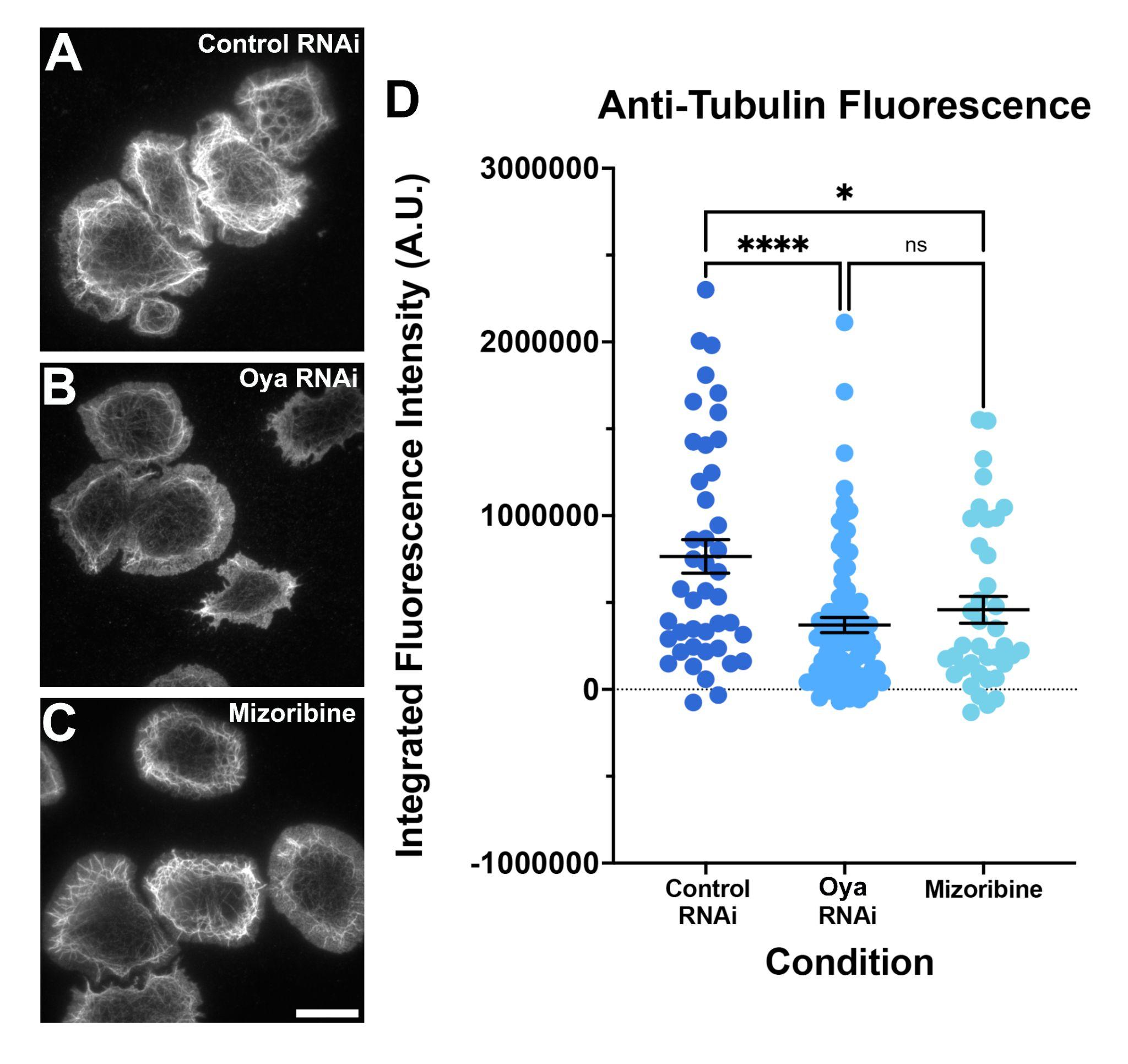


**Supplemental Figure 7. RNAi depletion of Oya leads to a decrease in tubulin fluorescence.** (A-C) S2R+ cells fixed and stained with anti-alpha tubulin antibody following treatment with (A) control RNAi, (B) Oya RNAi, or (C) 290µM Mizoribine. Images are presented using the same gray scale for direct comparison. Scale bar is 10 µm. (D) Quantification of the mean (± SEM) of the integrated tubulin fluorescence intensity for control RNAi (dark blue circles), Oya RNAi (medium blue circles), and 290 µM Mizoribine (cyan blue circles). There was a statistically significant decrease in integrated tubulin fluorescence following Oya RNAi treatment or treatment with Mizoribine as compared to control RNAi treated cells (**p-*value = 0.0143,*****p-*value <0.0001, one-way ANOVA with Tukey’s post-hoc analysis, N = 43-84 cells).
